# Design‐based properties of the nearest neighbor spatial interpolator and its bootstrap mean squared error estimator

**DOI:** 10.1111/biom.13505

**Published:** 2021-06-22

**Authors:** Lorenzo Fattorini, Marzia Marcheselli, Caterina Pisani, Luca Pratelli

**Affiliations:** ^1^ Department of Economics and Statistics University of Siena Siena Italy; ^2^ Naval Academy Livorno Italy

**Keywords:** environmental sampling, pointwise consistency, pseudopopulation bootstrap, spatial populations, uniform consistency

## Abstract

Nearest neighbor spatial interpolation for mapping continuous populations and finite populations of areas or units is approached from a design‐based perspective, that is, populations are fixed, and uncertainty stems from the sampling scheme adopted to select locations. We derive conditions for design‐based pointwise and uniform consistency of the nearest neighbor interpolators. We prove that consistency holds under certain schemes that are widely applied in environmental and forest surveys. Furthermore, we propose a pseudopopulation bootstrap estimator of the root mean squared errors of the interpolated values. Finally, a simulation study is performed to assess the theoretical results.

## 1 INTRODUCTION

The nearest neighbor (NN) criterion is widely adopted in several fields of statistical analysis. The criterion has the appealing property of being nonparametric: it is simply based on the supposition that data that are near in some sense tend to be similar. Among other areas, the NN criterion is adopted in pattern recognition and clustering problems, where an object of unknown category is classified in the same category of its nearest observed object (e.g., Devroye *et al.*, 1996; Bremner *et al.*, 2005; Everitt *et al.*, 2011), in nonparametric regression, where the predicted value of the variable of interest for a nonobserved unit is that attached to the nearest unit in the covariate space (e.g., Stone, 1977; Altman, 1992; Terrell and Scott, 1992), and in outlier and anomaly detection, where the larger the distance of an observation is to its NN, the more likely the observation is to be an outlier (e.g., Campos *et al.*, 2016).

The NN criterion has been adopted for spatial interpolation for a long time. In the case of continuous surfaces, given a set of *n* sampled locations for which the surface values have been recorded, the interpolated value at any other location is the value observed at the nearest sampled location. Practically speaking, the interpolated surface is a piecewise constant function assigning the value recorded at a sampled location to each location inside the Voronoi cell around the sampled location. The NN criterion can be extended to the interpolation of values in finite populations of areas or units.

Owing to its simplicity, mapping by NN interpolation constitutes a widely extended practice in many fields of research such as, among others, environmental and ecological surveys (e.g., Li and Heap, 2008), epidemiology and air quality (e.g., Wong *et al.*, 2004, and references therein), atmospheric sciences (e.g., Chen *et al.*, 2010), and high‐resolution imaging (e.g., Ashraf *et al.*, 2017).

Despite its large use, the NN spatial interpolator has been invariably adopted as a descriptive technique. From a model‐dependent perspective, Cressie (1993, section 5.9) classified descriptive mapping techniques for which no stochastic model is assumed and, as such, no uncertainty is associated, as “nonstochastic methods of spatial prediction.”

However, in recent years, another nonstochastic method of spatial prediction, that is, the widely applied inverse distance weighting (IDW) interpolator, has been approached from a design‐based perspective: the population to be interpolated is considered fixed, and uncertainty only stems from the probabilistic sampling scheme adopted to select locations. In IDW interpolation, the interpolated value is achieved as a convex combination of the values observed at sampled locations with weights decreasing with the distances to the location to be interpolated. Conditions ensuring design‐based asymptotic unbiasedness and consistency of the IDW interpolator have been proven for continuous populations (Fattorini *et al.*, 2018a), finite populations of areas partitioning a region (Fattorini *et al.*, 2018b), and finite populations of units located in a region (Fattorini *et al.*, 2019). For the three scenarios, design‐based asymptotic unbiasedness and consistency are achieved at the cost of supposing (*i*) some forms of smoothness of the survey variable throughout the study region; (*ii*) asymptotically balanced spatial sampling schemes; (*iii*) some mathematical properties of the distance functions adopted for weighting sampled observations; and (*iv*) some sort of regularities, such as in the shape of areas or in the enlargement of the populations of units, in the case of finite populations.

Regarding the distance function to adopt for weighting sample observations, it has been proven that negative powers of type $\phi \left(d\right)=d^{-\alpha }$, where *d* is a positive real number representing a distance and α is a positive real number, satisfy asymptotic unbiasedness and consistency of the IDW interpolator for α>2 (Fattorini *et al.*, 2018a; Fattorini *et al.*, 2018b; Fattorini *et al.*, 2019). Therefore, NN interpolator can be viewed as the limiting case of the IDW interpolator with weights of type $\phi \left(d\right)=d^{-\alpha }$ for α approaching infinity.

The purpose of this paper is to derive conditions sufficient to extend the asymptotic properties proven for the IDW interpolator to the NN interpolator. We do so in a unifying approach that includes the three types of spatial populations. Indeed, design‐based asymptotic unbiasedness and consistency of NN interpolator cannot be straightforwardly achieved from the inequalities regarding the IDW interpolator under the three scenarios as α approaches infinity. In this way, three different results would be obtained. On the other hand, because the NN interpolator only involves the nearest sample location, asymptotic results are achieved in a more direct and less cumbersome way and are jointly valid for the three types of spatial populations. Moreover, a pseudopopulation bootstrap approach is adopted to obtain a reliable, conservative estimator of the accuracy of the NN interpolator, for which no consistency result is presently available and uncertainty assessment has been traditionally neglected or has not yet gone beyond the simple application of leave‐one‐out or cross‐validation techniques (e.g., Chen *et al.*, 2010) without any theoretical investigation.

The paper is organized as follows. Notation and setting are introduced in Section 2. Section 3 contains some finite sample results useful for determining the asymptotic properties of the NN interpolator, which are detailed in Section 4. In Section 5, the asymptotic properties are proven to hold under familiar spatial schemes, and in Section 6, a pseudopopulation bootstrap estimator of the precision of the NN interpolator is proposed. A simulation study is described in Section 7, whereas the application of the NN interpolator for providing the forest map in a region in Casentino Valley is illustrated in Section 8. Finally, concluding remarks are given in Section 9. Supporting Information contains technical details and proofs, tables, and figures referring to the simulation study.

## 2 NOTATION AND SETTING

Denote by λ the Lebesgue measure on $R^{2}$ and by I(E) the indicator function of the event *E*. Let *Y* be a survey variable, and consider a study region *A* that is assumed to be a compact set of $R^{2}$. Moreover, let *f* be a measurable function defined on a Borelian subset *B* of *A*, with values on [0, *L*] and such that, for any Borelian subset *C* of *B*, $\int_{C}f\left(p\right)\mu \left(dp\right)$ yields the amount of *Y* in the region *C*, where μ is the Lebesgue measure λ under continuous populations and population of spatial areas, whereas it is the counting measure under a population of units. Then, in accordance with the features of spatial populations, there are the following three settings.

### 2.1 Continuous populations

*B* coincides with *A*, and *f* is the density of *Y*, that is, $\int_{C}f\left(p\right)\;\lambda \left(dp\right)$ is the amount of *Y* in *C*. Therefore, mapping necessitates the knowledge of f(p) for (almost) each p∈B.

### 2.2 Finite populations of spatial areas

*B* coincides with *A*, which is partitioned into *N* areas $a_{1},\text{…},a_{N}$, and $y_{j}$ is the amount of the survey variable *Y* within $a_{j}$. Therefore, mapping requires knowledge of $y_{j}$ for each j=1,…,N. As the area size $\lambda (a_{j})$ is usually known for each j=1,⋯,N, mapping actually requires knowledge of the density of *Y* within area *j*, $y_{j}/\lambda (a_{j})$, for each j=1,…,N, that is equivalent to the knowledge of the piecewise constant function

$$f\left(p\right)=\sum_{j=1}^{N}\frac{y_{j}}{\lambda (a_{j})}I\left(p\in a_{j}\right)$$

for each p∈B. In particular, $\int_{B}f\left(p\right)\lambda \left(dp\right)=y_{1}+\cdots +y_{N}$.

### 2.3 Finite populations of units

*B* is the set $\left\{p_{1},\cdots ,p_{N}\right\}$ of *N* unit locations, and $y_{j}=f\left(p_{j}\right)$ is the value of the survey variable for the unit *j*. Therefore, mapping requires the knowledge of $f(p_{j})$ for each j=1,⋯,N. It is worth noting that $\int_{B}f\left(p\right)\mu \left(dp\right)=y_{1}+\cdots +y_{N}$, where μ is the counting measure that yields mass 1 at every $p_{j}$ for j=1,⋯,N.

Let $P_{1},\cdots ,P_{n}$ be *n* random variables with values in *B* that represent the *n* locations selected from *B* by means of a probabilistic fixed‐size sampling scheme. In the case of continuous populations, $P_{1},\cdots ,P_{n}$ denote *n* locations selected in the continuum *B*, and $f\left(P_{1}\right),\cdots ,f\left(P_{n}\right)$ are the densities of *Y* recorded at those locations. In the case of finite populations of areas, $P_{1},\cdots ,P_{n}$ denote the centroids identifying the *n* sampled areas, and $f\left(P_{1}\right),\cdots ,f\left(P_{n}\right)$ are the densities recorded within the corresponding areas. Finally, in the case of finite populations of units, $P_{1},\cdots ,P_{n}$ denote the locations of *n* sampled units, and $f\left(P_{1}\right),\cdots ,f\left(P_{n}\right)$ are the values of *Y* for these units. The NN spatial interpolator $\hat{f}$ of *f* is

(1) $$\hat{f}\left(p\right)=I\left(Q_{p}\right)f\left(p\right)+\frac{I(Q_{p}^{c})}{\mathrm{Card}(H_{p})}\sum_{i\in H_{p}}f(P_{i})\;,\;p\in B,$$

where $Q_{p}={⋃}_{i=1}^{n}\left\{P_{i}=p\right\}$ and $H_{p}=\{i:\;∥P_{i}-p∥=\min_{h=1,\cdots n}∥P_{h}-p∥\}$.

In the continuous case, $\;Q_{p}$ has probability 0 and $\mathrm{Card}(H_{p})$ is equal to 1 almost surely, in such a way that (1) reduces almost surely to

(2) $$\hat{f}\left(p\right)=f\left(P_{i}\right)\;,\;p\in B,$$

where $∥P_{i}-p∥=\min_{h=1,\cdots n}∥P_{h}-p∥$.

In the case of finite populations of areas, $\hat{f}\left(p\right)=\hat{f}\left(b_{j}\right)$ for each $p\in a_{j}$, where $b_{j}$ denotes the centroid of the *j*th area, and

(3) $$\hat{f}\left(b_{j}\right)=I\left(Q_{b_{j}}\right)f\left(b_{j}\right)+\frac{I(Q_{b_{j}}^{c})}{\mathrm{Card}(H_{b_{j}})}\sum_{i\in H_{b_{j}}}f(P_{i})\;,\;\;\;\;j=1,\cdots ,N,$$

where $\mathrm{Card}(H_{b_{j}})$ may be greater than 1, as, for example, in the case of populations of regular polygons (e.g., pixels).

Finally, in the case of finite populations of units, the NN interpolator is

(4) $$\hat{f}\left(p_{j}\right)=I\left(Q_{p_{j}}\right)f\left(p_{j}\right)+\frac{I(Q_{p_{j}}^{c})}{\mathrm{Card}(H_{p_{j}})}\sum_{i\in H_{p_{j}}}f(P_{i})\;,\;\;\;j=1,\cdots ,N,$$

where, if units are settled on regular grids (e.g., net nodes), NNs may be more than 1.

Interpolator (1) is the limit of the IDW interpolator with distance function $d^{-\alpha }$

$${\hat{f}}_{\alpha }\left(p\right)=I\left(Q_{p}\right)f\left(p\right)+I\left(Q_{p}^{c}\right)\frac{\sum_{i=1}^{n}f\left(P_{i}\right){∥P_{i}-p∥}^{-\alpha }}{\sum_{i=1}^{n}{∥P_{i}-p∥}^{-\alpha }},$$

when α approaches infinity.

## 3 SOME FINITE SAMPLE RESULTS

We derive some results for *n* finite, to be subsequently exploited for determining the asymptotic properties of the NN interpolator.

Denote by ${∥\hat{f}-f∥}_{\infty }=\sup_{p\in B}\left|\hat{f}\left(p\right)-f\left(p\right)\right|$. In the following, without loss of generality, we suppose that

(5) $${∥\hat{f}-f∥}_{\infty }=\sup_{p\in D}\left|\hat{f}\left(p\right)-f\left(p\right)\right|$$

for a suitable countable subset D⊂B. Indeed, (5) is true when *f* is continuous or *B* is a countable set. For any δ>0 and p∈B, denote by

$$\Delta \left(p,\delta \right)=\sup_{p\in B:∥p-q∥\leq \delta }\left|f\left(q\right)-f\left(p\right)\right|$$

the largest jump of *f* in the δ‐ball of **p** and $\Delta \left(\delta \right)=\sup_{p\in D}\Delta \left(p,\delta \right)$ the largest jump on *D*.

Moreover, denote by $A_{i}\left(p,\delta \right)=\{∥P_{i}-p∥>\delta \}$ the event that the *i*th sampled location is outside the δ‐ball of **p**, in such a way that

$$A\left(p,\delta \right)=\cap_{i=1}^{n}A_{i}\left(p,\delta \right)=\cap_{i=1}^{n}\left\{∥P_{i}-p∥>\delta \right\}$$

is the event that no sampled location is within the δ‐ball of **p**.

Theorem 1 For any δ>0 and p∈B

(6) $$E\{|\hat{f}\left(p\right)-f\left(p\right)|\}\leq \Delta \left(p,\delta \right)+L\mathrm{Pr}\left\{A\left(p,\delta \right)\right\}.$$

Moreover, under condition (5)

(7) $$E\{{∥\hat{f}-f∥}_{\infty }\}\leq \Delta \left(\delta \right)+L\mathrm{Pr}\left\{\underset{p\in D}{⋃}A(p,\delta )\right\}.$$

Both the inequalities highlight that expectations of absolute errors are bounded by the sum of two terms: the first depending on the roughness of *f* and the second depending on the sampling design. Therefore, precise interpolation takes hold when both terms are small. If *f* is continuous at **p** or on the whole *B*, then the first term on the right‐hand sides of (6) and (7) approaches zero with δ, and accordingly, the precision of the interpolation depends on the features of the sampling design throughout the second terms. Practically speaking, the sampling scheme should be able to ensure a spatial balance, that is, to evenly spread sampled locations in such a way that a location, in the case of (6), or any location on the whole *B*, in the case of (7), is likely to have neighboring locations sampled. In turn, regarding the right‐hand side of (6), the second term can be bounded on the basis of the random variable $Z\left(p,\delta \right)=\sum_{i=1}^{n}I\left\{A_{i}^{c}\left(p,\delta \right)\right\}$ representing the number of sampled locations falling in the δ‐ball of **p**.

Theorem 2 For any fixed *n* and for any δ>0 and p∈B

(8) $$\begin{array}{ccc} & & \mathrm{Pr}\left\{A\left(p,\delta \right)\right\}=\mathrm{Pr}\left\{Z\left(p,\delta \right)=0\right\}\leq \frac{1}{\sum_{i=1}^{n}\mathrm{Pr}\{A_{i}^{c}\left(p,\delta \right)\}}\\ & & \;+\;\sup_{h\neq i=1,\cdots ,n}{\left[\frac{\mathrm{Pr}\{A_{i}^{c}\left(p,\delta \right)\cap A_{h}^{c}\left(p,\delta \right)\}}{\mathrm{Pr}\left\{A_{i}^{c}\left(p,\delta \right)\right\}\mathrm{Pr}\left\{A_{h}^{c}\left(p,\delta \right)\right\}}-1\right]}^{+}. \end{array}$$

The precision of the NN interpolator deteriorates where discontinuities are present. However, the precision of the whole map is preserved if these discontinuities, as usual in practical situations, occur for sets of measure zero. Indeed, in this case, the mean integrated absolute error

(9) $$MIAE\left(\hat{f}\right)=\int_{B}E\{|\hat{f}\left(p\right)-f\left(p\right)|\}\lambda \left(dp\right)$$

strictly depends on $\int_{B}\mathrm{Pr}\{A(p,\delta )\}\lambda \left(dp\right)$ that, in turn, will be small if the second term of (6) is small due to the effectiveness of the sampling design.

More compelling results are achieved if we suppose *f* to be Lipschitz continuous at **p**, that is, if |f(q)−f(p)|≤β∥p−q∥ for each q∈B, where β>0 is the Lipschitz constant. In this case, taking $\delta =tn^{-1/2}$ for any t>0, from inequalities (6) and (7), it follows that

(10) $$E\{|\hat{f}\left(p\right)-f\left(p\right)|\}\leq \beta tn^{-1/2}+L\mathrm{Pr}\left\{A\left(p,tn^{-1/2}\right)\right\},$$

(11) $$E\left({∥\hat{f}-f∥}_{\infty }\right)\leq \beta tn^{-1/2}+L\mathrm{Pr}\left\{\underset{p\in D}{⋃}A(p,tn^{-1/2})\right\},$$

respectively. The previous inequalities will be useful in investigating the asymptotic properties of interpolator (1) under suitable spatial schemes.

## 4 ASYMPTOTIC RESULTS

To achieve design‐based asymptotic unbiasedness and consistency of (1), the following three asymptotic scenarios are considered. All of them refer to the infill asymptotics paradigm (Cressie, 1993) and have already been exploited in Fattorini *et al.* (2018a), Fattorini *et al.* (2018b), and Fattorini *et al.* (2019).

In the case of continuous populations, a sequence of fixed‐size designs to select samples of increasing size on the fixed subset *B* is assumed. In particular, for any natural number *k*, a fixed‐size design selecting a sample of $n_{k}$ locations $P_{k,1},\cdots ,P_{k,n_{k}}$ from *B* is considered, with $n_{k}\to \infty$ as *k* increases, and for each p∈B, ${\hat{f}}_{k}\left(p\right)$ is the NN interpolator (2) of f(p).

In the case of finite populations of areas, *B* is fixed, and for any natural number *k*, *B* is partitioned into $N_{k}$ units $a_{k,1},\cdots ,a_{k,N_{k}}$ with centroids $b_{k,1},\cdots ,b_{k,N_{k}}$, where, as *k* increases, $N_{k}↑\infty$ and all the units decrease in size such that $\sup_{j=1,\cdots ,N_{k}}\mathrm{diam}\left(a_{k,j}\right)\to 0$. Then, a sequence of fixed‐size designs is considered to select samples of ${n_{k}<N}_{k}$ areas identified by their centroids $P_{k,1},\cdots ,P_{k,n_{k}}$, with $n_{k}\to \infty$. Therefore, referring to the *k*th partition, for each $p\in a_{k,j}$ and $j=1,\cdots ,N_{k}$, ${\hat{f}}_{k}\left(p\right)={\hat{f}}_{k}\left(b_{k,j}\right)$ is the NN interpolator (3) of the piecewise constant function $f_{k}\left(p\right)$.

Finally, in the case of finite populations of units, as is customary in the finite population asymptotic framework (Särndal *et al.*, 1992), let $V=\{p_{1},p_{2},\cdots \}$ be an infinite sequence of points onto *A*. A sequence $\left\{B_{k}\right\}$ of populations is considered where *B*_1_ consists of the first *N*_1_ points from V, *B*_2_ consists of the first *N*_2_ points from V with $N_{2}>N_{1}$, and so on, in such a way that $\left\{B_{k}\right\}$ turns out to be a sequence of nested populations of increasing sizes. Finally, suppose a sequence of fixed‐size designs to select a sample of size $n_{k}$ of units identified by the locations $P_{k,1},\cdots ,P_{k,n_{k}}$ from $B_{k}$ with $n_{k}\to \infty$. Therefore, referring to the *k*th population, for each $p_{j}\in B_{k}$, ${\hat{f}}_{k}\left(p_{j}\right)$ is the NN interpolator (4) of $f(p_{j})$.

A unique definition of design‐based consistency can be given for all the asymptotic scenarios. In particular, NN interpolator (1) is pointwise design consistent at $p\in B_{k}$ if for any ε>0

$$\lim_{k\to \infty }\mathrm{Pr}\{|{\hat{f}}_{k}\left(p\right)-f_{k}\left(p\right)\left|>\varepsilon \right\}=0,$$

and it is uniformly consistent if

$$\lim_{k\to \infty }\mathrm{Pr}\left\{{∥{\hat{f}}_{k}-f_{k}∥}_{\infty }>\varepsilon \right\}=0,$$

where for any *k*, $B_{k}=B$ in the cases of continuous and area populations and $f_{k}=f$ in the cases of continuous and unit populations. Because in all cases, the $f_{k}$s are bounded with values in [0, *L*], pointwise or uniform design consistency also entails pointwise or uniform design asymptotic unbiasedness.

From inequalities (6) and (7), taking $\delta_{k}=tn_{k}^{-1/2}$ for any t>0, the first terms of (6) and (7) approach 0 with $\delta_{k}$. Therefore, pointwise and uniform consistency of (1) is obviously achieved if the sequence of sampling designs ensures that, for any ε>0, there exist a real t>0 and an integer *k*_0_ such that

(12) $$\mathrm{Pr}\left\{A_{k}\left(p,tn_{k}^{-1/2}\right)\right\}<\varepsilon ,\;\;\;\forall k>k_{0}$$

or if

(13) $$\mathrm{Pr}\left\{\underset{p\in D_{k}}{⋃}A_{k}\left(p,tn_{k}^{-1/2}\right)\right\}<\varepsilon ,\;\;\;\forall k>k_{0},$$

respectively, where $A_{k}\left(p,\delta \right)=\cap_{i=1}^{n_{k}}\left\{∥P_{k,i}-p∥>\delta \right\}$ and $D_{k}$ is a suitable countable subset of $B_{k}$.

## 5 ASYMPTOTIC BEHAVIOR UNDER FAMILIAR SPATIAL SCHEMES

Sampling locations from a continuous population can be performed by uniform random sampling (URS), that is, the random and independent selection of *n* locations. We prove that under URS, condition (12) invariably holds, ensuring pointwise consistency of (1). URS is probably the most straightforward scheme but may lead to uneven surveying of *B*.

Many schemes are available for sampling spatial locations from a continuum that are able to achieve even coverage of the study region, so‐called spatial balance. Spatial balance can be obtained by the use of quite complex, explicitly tailored schemes (e.g., Stevens and Olsen, 2004; Lister and Scott, 2009). Alternatively, spatial balance can be readily obtained by simple schemes involving the tessellation of the study region into *n* regular polygons and the random or systematic selection of one location per polygon. The two schemes are referred to as tessellation stratified sampling (TSS) and systematic grid sampling (SGS), respectively, and are widely applied in environmental surveys, especially forest surveys at large scale (e.g., Tomppo *et al.*, 2010). Indeed, these schemes have the appealing property that, for a suitable t>0, they ensure $\mathrm{Pr}\{{⋃}_{p\in D_{k}}A_{k}\left(p,tn_{k}^{-1/2}\right)\}=0$ and therefore the uniform consistency of the NN interpolator. Moreover, from (10) or (11), under the Lipschitz condition at **p** or for the whole *B*, $E\{|{\hat{f}}_{k}\left(p\right)-f_{k}\left(p\right)|\}$ or $E\{{∥{\hat{f}}_{k}-f_{k}∥}_{\infty }\}$ are $O(n_{k}^{-1/2})$, that is, consistency occurs at a rate of $n_{k}^{-1/2}$.

Similarly, many schemes are available for sampling finite populations of areas and units that are able to achieve spatial balance. Also, in these cases, spatial balance can be obtained by the use of explicitly tailored schemes (see, e.g., Grafström and Tillé, 2013, and references therein) or by the use of simple schemes that involve the stratification of the population into *n* regular blocks of contiguous areas or units and the random or systematic selection of one area or one unit per block. The two schemes are referred to as one‐per‐stratum stratified sampling (OPSS) and systematic sampling (SYS), respectively, and have long history in the statistical literature (e.g., Breidt, 1995). Moreover, in this case, the two schemes ensure that, for a suitable t>0, $\mathrm{Pr}\{{⋃}_{p\in D_{k}}A_{k}\left(p,tn_{k}^{-1/2}\right)\}=0$. Therefore, they ensure uniform consistency and, under the Lipschitz condition, consistency occurs at a rate of $n_{k}^{-1/2}$.

Regarding the concept of spatial balance in finite populations of spatial areas and units, Stevens and Olsen (2004) link this concept to the NN structure, proposing to quantify the spatial balance of a sample by the variance of the sums of inclusion probabilities of those units lying in the Voronoi polygons determined by the sample units (on the issue, see also Grafström *et al.*, 2014). However, it should be noted that the asymptotical spatial balance involved by condition (12) or (13) can be achieved even by schemes not explicitly intended to achieve spatial balance. One of these schemes is the so‐called 3P sampling (from the acronym of probability proportional to prediction). Indeed, regarding populations of units, their mapping is precluded in the absence of population lists and locations. Therefore, mapping is unfeasible in forest and environmental surveys where populations are communities scattered over large areas without any possibility of having lists. Probably, the unique relevant case in which the mapping of natural populations is possible is under 3P sampling. The scheme is a variation of Poisson sampling: all the units in the population are visited by a crew of experts (and hence listed and mapped), a prediction $x_{j}$ for the value of the survey variable is given for each unit *j*, and units are independently included in the sample with probabilities $\pi_{j}=x_{j}/L^{*}$, where $L^{*}>L$ is chosen to ensure $\pi_{j}\leq 1$ for each *j* and adequate values of expected sample size (e.g., Gregoire and Valentine, 2008). Because prediction errors $e_{j}=y_{j}-x_{j}$ are known for each sampled unit, Fattorini *et al.* (2019) suggest interpolating the $e_{j}$s instead of the $y_{j}$s and then achieving the interpolated *Y*‐values by means of ${\hat{y}}_{j}=x_{j}+{\hat{e}}_{j}$ for each j∈B. Even if prediction errors can take negative values, they are bounded by *L* in such a way that all the consistency results continue to hold. The mapping improvement with respect to the direct interpolation of the $y_{j}$s has been investigated by Fattorini *et al.* (2020) and has been proven to be relevant.

To prove the pointwise consistency of (1) under 3P sampling, analogously to Fattorini *et al.* (2019), we further assume the following condition: $V=\{p_{1},p_{2},\text{…}\}$ is regular, that is, for any $p_{j}\in V$ and for any natural number *m*, there exist a real number t>0 and an integer *k*_0_ such that

(14) $$\mathrm{Card}\left\{B_{j}\left(tN_{k}^{-1/2}\right)\cap B_{k}\right\}>m,\;\;\;\forall k>k_{0},$$

where $B_{j}\left(\delta \right)$ is the set of units of *V* in the δ‐ball of unit *j*. Condition (14) requires that the populations in the sequence increase in such a way that, for a sufficiently large *k*, any unit has many neighboring units around it. Under this condition, we prove that 3P sampling ensures the consistency of (1) when the inclusion probabilities $\pi_{j,k}$ are invariably greater than a threshold $\pi_{0}>0$ for any $j\in B_{k}$ and any *k*. A lower bound for *Y* is common in forest and environmental surveys in which units with *Y*‐values (e.g., tree height or basal area) smaller than a given threshold l>0 are not considered in the population such that $\pi_{0}=l/L^{*}$.

## 6 PSEUDOPOPULATION BOOTSTRAP ESTIMATION OF PRECISION

Mashreghi *et al.* (2016) provide extended surveys of the bootstrap methods adopted for design‐based inference. Among them, the pseudopopulation bootstrap is based on constructing a pseudopopulation likely to resemble the true population from which bootstrap samples are selected using the same sampling scheme adopted in the survey. In this setting, the key problem is to reconstruct pseudopopulations able to mimic the characteristics of the unknown populations in such a way that the bootstrap distribution of a statistic resembles the true distribution with bootstrap mean squared error approaching the true one (e.g., Quatemberg, 2016). Accordingly, to estimate the precision of (1), we use the estimated maps as pseudopopulations from which bootstrap samples are selected by means of the same spatial scheme adopted to select the original sample. If estimated maps converge to true ones, the bootstrap distributions of the NN interpolator achieved from resampling from these maps should converge to the true distributions, also providing reliable estimators of their mean squared errors.

Let $\hat{f}\left(B\right)=\left\{\hat{f}\left(p\right),p\in B\right\}$ be the estimated map based on ${f(P}_{1}),\cdots ,f\left(P_{n}\right)$. For each p∈B, the pseudopopulation bootstrap estimator of the root mean squared error of $\hat{f}\left(p\right)$ is

(15) $${\hat{rmse}}_{M}^{*}\left(p\right)={\left[\frac{1}{M}\sum_{m=1}^{M}{\left\{{\hat{f}}_{m}^{*}\left(p\right)-\hat{f}\left(p\right)\right\}}^{2}\right]}^{1/2},$$

where *M* is the number of bootstrap samples and ${\hat{f}}_{m}^{*}\left(p\right)$ is the bootstrapped value of the NN interpolator at p∈B based on $\hat{f}\left(P_{1,m}^{*}\right),\cdots ,\hat{f}\left(P_{n,m}^{*}\right)$ (obtained from the estimated map $\hat{f}\left(B\right)$), that is, for any p∈B and m=1,…,M

(16) $${\hat{f}}_{m}^{*}\left(p\right)=I\left(Q_{p,m}^{*}\right)\hat{f}\left(p\right)+\frac{I(Q_{p,m}^{*c})}{\mathrm{Card}(H_{p,m}^{*})}\sum_{i\in H_{p,m}^{*}}\hat{f}\left(P_{i,m}^{*}\right),$$

where $P_{1,m}^{*},\cdots ,P_{n,m}^{*}$ are the locations selected in the *m*th bootstrap resampling, $Q_{p,m}^{*}=\cup_{i=1}^{n}\left\{P_{i,m}^{*}=p\right\}$ and $H_{p,m}^{*}=\{i:\;∥P_{i,m}^{*}-p∥=\min_{h=1,\cdots n}∥P_{h,m}^{*}-p∥\}$.

We obtain a finite sample result about (15) supposing two further conditions: (i) for a given sample size *n*, the sampling design ensures the existence of a δ>0 such that

(17) Pr{A(p,δ)}=0,

(ii) there exist a vector $a\in R^{2}$, a≠0 and a function q↦o(∥q−p∥) negligible with respect to ∥q−p∥, such that

(18) $$f\left(P_{i}\right)=f\left(p\right)+\left\langle a,P_{i}-p\right\rangle +o(∥P_{i}-p∥),\;\;i=1,\text{…},n.$$

Theorem 3 For a given *n*, under conditions (17) and (18) and for *M* large enough,

(19) $$\frac{E\{{\hat{rmse}}_{M}^{*}\left(p\right)\}}{E{\left[{\left\{\hat{f}\left(p\right)-f\left(p\right)\right\}}^{2}\right]}^{1/2}}\leq 3.$$

The requirement of *M* being large enough can be readily satisfied by increasing the computational effort. Condition (17) is less restrictive than condition (12), and it holds for all the sampling schemes discussed in Section 5 that ensure the pointwise consistency of the NN interpolator. On the other hand, condition (18) requires that in the case of continuous populations and finite populations of areas, *f* is differentiable at **p** with ∇f(p)≠0, whereas this requirement is not necessary for finite populations of points. Practically speaking, Theorem 3 states that, under suitable conditions, the pseudopopulation bootstrap estimator (15) tends to be conservative, with its expectation being at most three times greater than the true root mean squared error. Even if the result may induce one to suspect substantial overestimation that may mask the effectiveness of interpolation, 3 is just a threshold limiting possible overestimation. Finally, for n→∞ and for *M* sufficiently large, the consistency of (15) is obvious from (19). Indeed, from (19), for any *n*, it holds that (15) is bounded by three times the true root mean squared error. However, owing to the consistency of the NN interpolator under the required conditions, the true root mean squared error tends to 0 as *n* increases, so that (15) tends to 0 a fortiori.

## 7 SIMULATION STUDIES

We consider three artificial surfaces on the unit square *A* to generate continuous populations, finite populations of areas, and finite populations of units, referred to as surface 1, surface 2, and surface 3, and, respectively, defined at any location $p=(p_{1},p_{2})$ as

$$f\left(p\right)=\frac{C_{1}}{2}\left(\sin^{2}p_{1}+\cos^{2}p_{2}+p_{1}\right),\;\;\;\;f\left(p\right)=C_{2}\left(\sin3p_{1}\;\sin^{2}{3p}_{2}\right),$$

$$f\left(p\right)=\left\{\begin{array}{cc} C_{3}p_{1}p_{2} & \mathrm{if}\;\min(p_{1},p_{2})\leq 1/2\\ C_{3}\left(1+p_{1}\right)p_{2} & \mathrm{otherwise}. \end{array}\right.$$

where the constants *C*_1_, *C*_2_, and *C*_3_ ensure a maximum value L=10. The three surfaces are represented in Web Figure 3 in the Supporting Information.

Regarding continuous populations, the three surfaces were taken as population values on B=A. Sampling was performed selecting n=16,36,64,100 locations on *B* by means of URS, TSS, and SGS. The last two schemes were performed by partitioning *B* into 4 × 4, 6 × 6, 8 × 8, and 10 × 10 grids of equal‐sized quadrats and selecting a location in each quadrat.

Regarding finite populations of areas, the three surfaces were used to generate the *Y*‐values within the areas. For each surface, four populations of N=100,400,900,1600 areas were constructed by partitioning B=A into grids of 10 × 10, 20 × 20, 30 × 30, 40 × 40 quadrats and taking the integrals of the surface within quadrats as population values from which densities are derived. Sampling was performed by selecting n=0.1N quadrats by means of simple random sampling without replacement (SRSWOR), OPSS, and SYS. The last two schemes were performed by partitioning grids into blocks of 2 × 5 contiguous quadrats and selecting one quadrat per block. Regarding unit populations, three nested populations of 500, 1000, and 1500 units were located on *A* in accordance with four spatial patterns referred to as regular, random, trended, and clustered patterns. For the regular pattern, populations were constructed by independently generating the first 500 locations at random but discarding those having distances smaller than $0.5\times 500^{-1/2}$ to those previously generated, then adding 500 further locations at random but discarding those with distances smaller than ${0.5\times 1000}^{-1/2}$ to those previously generated, and finally randomly adding a further 500 locations but discarding those having distances smaller than ${0.5\times 1500}^{-1/2}$ to those previously generated. For the random pattern, populations were constructed by independently generating 1500 locations at random on *A* and then assigning the first 500 to the smaller population, the first 1000 to the second, and all of them to the largest. For the trended pattern, populations were constructed by independently generating 1500 pairs of random numbers $u_{1},u_{2}$ uniformly distributed on [0,1], performing the transformation $\left(1-u_{1}^{2},1-u_{2}^{2}\right)$ to determine locations, and then assigning the first 500 locations to the smallest population, the first 1000 to the second, and all of them to the largest. For the clustered pattern, populations were constructed by independently generating 10 cluster centeres at random on *A* and assigning 50 locations to each cluster generated from a spherical normal distribution centered at the cluster center with variance 0.025, adding a further 50 locations to each cluster from the same distribution and finally adding a further 50 locations to each cluster from the same distribution. Points falling outside *A* were discarded and newly generated.

The three surfaces were used for assigning the *Y*‐values in the populations. 3P sampling with $L^{*}=50$ was adopted to select units. Units with *Y*‐values smaller than l=4 were discarded from the populations to ensure a lower bound of $\pi_{0}=0.08$ for the inclusion probabilities. Expert predictions for the $y_{j}$s were generated using the relationship $x_{j}=a+by_{j}$ with b=1−ρ(L+l)/(L−l) and a=(1+ρ)l−bl in accordance with Fattorini *et al.* (2020), assuming that predictions increased linearly with *Y*‐values with a maximum error rate ρ=0.10 occurring at the extremes. The predictions, joined with the *l* and $L^{*}$ choices, ensured an expected sampling fraction of approximately 12% in all cases.

For each combination of population, sampling scheme, and sample size, sampling was replicated R=10,000 times. At each simulation run *r*, the estimated map ${\hat{f}}_{r}\left(p\right)$, p∈B was obtained from (1), and M=1000 bootstrap samples were independently selected from the estimated map adopting the same scheme adopted to select the original sample to compute the bootstrap root mean squared error of ${\hat{f}}_{r}\left(p\right)$ for each p∈B by means of Equation (15). In the case of continuous populations, mapping was performed by computing ${\hat{f}}_{r}\left(p\right)$ for a regular grid of 100 × 100 locations on *B*.

Based on the Monte Carlo distributions, Web Tables 1–9 of the Supporting Information report the minima, averages, and maxima of the absolute bias and root mean squared error of (1) and of the ratio of the expectation of bootstrap root mean squared error (15) to the true value of root mean squared error. Web Figures 4–33 of the Supporting Information show the spatial patterns of these performance indicators.

Simulation results confirm the theoretical findings.

For populations generated from the continuous surfaces 1 and 2, a sharp decrease in the minima, averages, and maxima of absolute bias values and of mean squared errors occurs as the sample size (continuous populations) or population and sample sizes (finite populations of areas and units) increase.

For continuous populations and finite populations of areas generated from surface 3, with discontinuity at the internal edges of the upper‐right quadrant of the unit square, decreases occur only for minima and averages, whereas maxima decrease more slowly. However, as mean squared error averages can be viewed as the empirical counterparts of mean integrated absolute error (9), the consistency of maps is preserved overall, notwithstanding discontinuities. That is also apparent from Web Figures 10–12 and 19–21 of the Supporting Information.

For finite populations of units generated from surface 3, the slow decrease in maxima disappears. In these cases, maxima decrease as the minima and averages because discontinuities in *Y*‐values are absorbed by the corresponding predictions, providing negligible jumps in prediction errors. On the whole, decreases that occurred in finite populations of units are less marked than those that occurred in continuous populations and finite populations of areas because the use of prediction errors provides formidable gains in precision even for small population and sample sizes, leaving limited room for improvement as sizes increase.

Regarding bootstrap root mean squared errors, the ratios of their expectations to the true root mean squared error are, with very few exceptions, invariably greater than one on average and tend to asymptotically increase toward 1.2–1.5. Minima of this ratio evidence the presence of underestimation that tends to decline asymptotically, thus confirming the conservative nature of the bootstrap root mean squared error estimator. That is apparent from the web figures, where the lighter zones, corresponding to underestimation, become continually decrease as population and sample sizes increase. Maxima of this ratio evidence the possibilities of large overestimation that occur in presence of discontinuities (surface 3) and especially under systematic schemes. In the other cases, the maxima rarely exceed 2.

## 8 CASE STUDY

The NN interpolator was adopted to provide the forest map in a region *A* of 4900 ha located in Casentino Valley, the Eastern part of the Tuscany Region (Central Italy). The forest land is mainly characterized by mountainous beech forest, coniferous forest, and thermophilous deciduous forest. The climate is temperate‐humid: mean annual temperature is approximately 10^o^C, and total annual rainfall is greater than 1000 mm, with an average of more than 55 mm in the summer months (June–August). The forest grows on sandy‐loamy or loamy soils, rich in humus on the surface horizons. Soil depth varies. The slopes are generally steep or very steep. The area is characterized by forest exploitation, and thus, the estimation of a map for the dichotomous survey variable forest/not‐forest land is essential for analyzing the effects of human activities. Specifically, the function *f* related to the dichotomous survey variable is such that, for each p∈A, f(p) is equal to 1 if **p** is in the forest and equal to 0 otherwise.

**FIGURE 1:**
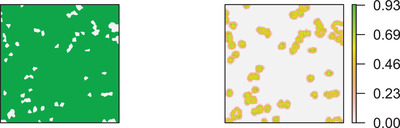
(a) Estimated map of forest (not‐white) and not‐forest (white) presence (left). (b) Map of estimated precision (right). This figure appears in color in the electronic version of this article, and any mention of color refers to that version

The survey was performed in 2013, and sample locations were selected by means of TSS. The study region was partitioned into 1225 quadrats of 200 m side, and a location was randomly selected within each quadrat. Each sample location was assigned a value of 1 if lying in the forest and a value of 0 otherwise. Based on the 1225 sample locations, the NN interpolator (2) was adopted to estimate f(p) for each location in the regular network of 1000 × 1000 nodes within *A*. The large number of locations at which estimation was performed ensured a good resolution of the resulting map displayed in Figure 1(a), which evidences the massive presence of forested land in the study region, notwithstanding the intensive management.

Moreover, at each location of the network, we also estimated the root mean squared error based on B=1000 bootstrap samples of size 1225 selected from the pseudopopulation of the interpolated values using the same sampling scheme adopted to select the original sample. The map of bootstrap root mean squared errors is reported in Figure 1(b), which shows that uncertainty increases when there is a change from forest to not‐forest, as expected owing to the theoretical findings. Indeed, when the dichotomous variable forest/not‐forest jumps from 1 to 0 along forest edges, *f* exhibits discontinuities, and the precision of the NN interpolator deteriorates.

## 9 CONCLUSIONS

Conditions for design‐based consistency of maps achieved from NN interpolation for continuous populations and finite populations of areas or units are given. Beyond the condition on the smoothness of surfaces generating populations, consistency conditions only regard the features of the sampling schemes adopted to select points, areas, or units. The use of TSS or SGS in continuous populations, the use of OPSS and SYS in finite populations of areas and of 3P sampling in finite populations of units ensures consistency. The focus on these schemes is not incidental; they nearly cover the range of possibilities to be adopted in forest and environmental surveys, as naturalists tend to avoid complex schemes, preferring schemes that are simple to be implemented and achieve spatial balance. Therefore, the achieved consistency results add statistical rigor to an interpolation technique widely used in environmental surveys with descriptive aims without attempting inference.

In addition, apart from when NN interpolation is applied in finite populations of regular polygons or finite populations of units located on a regular network, when NNs may be more than one and the interpolator is the convex combination of sample data recorded at these nearest locations, in most cases, there is only a single neighbor. Thus, the interpolated values have the same support as the *Y* variable, even when the support is discrete. This allows the application of NN interpolation for constructing maps of dichotomous 0–1 variables as in the case study, where a map of forest/not‐forest land is obtained. Relevantly, the resulting surfaces are piecewise constant with discontinuities along borders of 0 measure, and as such are Lipschitizian almost everywhere, thus providing consistency at a rate of $n^{-1/2}$ under suitable stratified and SYS schemes. This is of practical importance in certain applications such as land use and land cover mapping, a vital issue in the present period of substantial deforestation and urban sprawl. Indeed, the accuracy of land cover maps and its reliable estimation, which has a long tradition in the literature (e.g., Stehman and Czaplewski, 1998; Stehman, 2009; and references therein) can be straightforwardly and rigorously addressed in a design‐based approach by means of NN interpolation and bootstrap estimation of mean squared errors. Even if in this case, the survey variable is of multivariate nature being equal to the *k*th vector of the standard basis of $R^{K}$ when the point is in the *k*th land category (k=1,…,K), the consistency results continue to hold marginally for each map of the *K* land categories.

Finally, when auxiliary variables are available for the whole study region at little or no cost, as usually occurs in forest inventories (e.g., Opsomer *et al.*, 2007), NN interpolation can be performed in the auxiliary space, that is, the interpolated value at a location is the value observed at the sample location that is nearest in the auxiliary space. This intriguing idea has been empirically investigated by Grafström *et al.* (2014), achieving promising results that, however, necessitate further theoretical investigations to be fully confirmed.

## Supporting information

Web Appendices containing technical details and proofs, tables, and figures referenced in Sections 3, 5, 6, and 7 are available with this paper at the Biometrics website on the Wiley Online Library. In addition to this, the Fortran code implementing the simulation study and the case study are available at the Biometrics website on Wiley Online Library.

Supporting Information

## Data Availability

The data that support the findings in this paper are available in the Supporting Information of this article.
