# Supplementary material for: Design‐based properties of the nearest neighbor spatial interpolator and its bootstrap mean squared error estimator
Source: Biometrics. 2021 Jun 22;78(4):1454–63. doi: 10.1111/biom.13505 (PMC10078774; doi:10.1111/biom.13505)
Supplement: Supplementary file 1 — Web Appendices containing technical details and proofs, tables, and figures referenced in Sections 3, 5, 6, and 7 are available with this paper at the Biometrics website on the Wiley Online Library. In addition to this, the Fortran code implementing the simulation study and the case study are available at the Biometrics website on Wiley Online Library. [file BIOM-78-1454-s002.pdf]

Supporting information for "Design-based properties of the nearest neighbour spatial interpolator and its bootstrap mean squared error estimator"

Lorenzo Fattorini, Marzia Marcheselli, Caterina Pisani
Department of Economics and Statistics, University of Siena
and
Luca Pratelli
Naval Academy, Livorno

Web Appendix A. Proof of Theorem 1

$$
\begin{aligned}
|\hat{f}(\mathbf{p}) - f(\mathbf{p})| &= |I(Q_{\mathbf{p}})f(\mathbf{p}) + \frac{I(Q_{\mathbf{p}}^c)}{\text{Card}(H_{\mathbf{p}})} \sum_{i \in H_{\mathbf{p}}} f(\mathbf{P}_i) - f(\mathbf{p})| \\
&= |\frac{I(Q_{\mathbf{p}}^c)}{\text{Card}(H_{\mathbf{p}})} \sum_{i \in H_{\mathbf{p}}} f(\mathbf{P}_i) - \{1 - I(Q_{\mathbf{p}})\} f(\mathbf{p})| \\
&= |\frac{I(Q_{\mathbf{p}}^c)}{\text{Card}(H_{\mathbf{p}})} \sum_{i \in H_{\mathbf{p}}} f(\mathbf{P}_i) - I(Q_{\mathbf{p}}^c) f(\mathbf{p})| \\
&= I(Q_{\mathbf{p}}^c)|\frac{1}{\text{Card}(H_{\mathbf{p}})} \sum_{i \in H_{\mathbf{p}}} f(\mathbf{P}_i) - f(\mathbf{p})| \\
&= I(Q_{\mathbf{p}}^c)|\frac{1}{\text{Card}(H_{\mathbf{p}})} \sum_{i \in H_{\mathbf{p}}} f(\mathbf{P}_i) - \frac{1}{\text{Card}(H_{\mathbf{p}})} \sum_{i \in H_{\mathbf{p}}} f(\mathbf{p})| \\
&= I(Q_{\mathbf{p}}^c)|\frac{1}{\text{Card}(H_{\mathbf{p}})} \sum_{i \in H_{\mathbf{p}}} \{f(\mathbf{P}_i) - f(\mathbf{p})\}| \\
&\leq \frac{I(Q_{\mathbf{p}}^c)}{\text{Card}(H_{\mathbf{p}})} \sum_{i \in H_{\mathbf{p}}} |f(\mathbf{P}_i) - f(\mathbf{p})| \\
&= \frac{I(Q_{\mathbf{p}}^c)}{\text{Card}(H_{\mathbf{p}})} \sum_{i \in H_{\mathbf{p}}} [I\{A_i(\mathbf{p}, \delta)\} + I\{A_i^c(\mathbf{p}, \delta)\}] |f(\mathbf{P}_i) - f(\mathbf{p})| \\
&= \frac{I(Q_{\mathbf{p}}^c)}{\text{Card}(H_{\mathbf{p}})} \sum_{i \in H_{\mathbf{p}}} I\{A_i(\mathbf{p}, \delta)\} |f(\mathbf{P}_i) - f(\mathbf{p})| \\
&+ \frac{I(Q_{\mathbf{p}}^c)}{\text{Card}(H_{\mathbf{p}})} \sum_{i \in H_{\mathbf{p}}} I\{A_i^c(\mathbf{p}, \delta)\} |f(\mathbf{P}_i) - f(\mathbf{p})|.
\end{aligned}
\tag{A.1}
$$

Because the quantity $I\{A_i^c(\mathbf{p}, \delta)\}|f(\mathbf{P}_i) - f(\mathbf{p})|$ is equal to 0 if $I\{A_i^c(\mathbf{p}, \delta)\} = 0$ and is equal to $|f(\mathbf{P}_i) - f(\mathbf{p})|$ if $I\{A_i^c(\mathbf{p}, \delta)\} = 1$, ie, if the ith sampled observation is in the δ-ball of $\mathbf{p}$, then the quantity is invariably smaller than $\Delta(\mathbf{p}, \delta)$. Therefore, from (A.1) it follows that

$$
\begin{aligned}
|\hat{f}(\mathbf{p}) - f(\mathbf{p})| &\leq \frac{L}{\text{Card}(H_{\mathbf{p}})} \sum_{i \in H_{\mathbf{p}}} I\{A_i(\mathbf{p}, \delta)\} + \frac{1}{\text{Card}(H_{\mathbf{p}})} \sum_{i \in H_{\mathbf{p}}} \Delta(\mathbf{p}, \delta) \\
&= L \max_{i \in H_{\mathbf{p}}} I\{A_i(\mathbf{p}, \delta)\} + \Delta(\mathbf{p}, \delta).
\end{aligned} \tag{A.2}
$$

Because $H_{\mathbf{p}}$ is constituted by sampled locations having the same distance to $\mathbf{p}$, then the $I\{A_i(\mathbf{p}, \delta)\}$s are equal for all $i \in H_{\mathbf{p}}$. Accordingly, $\max_{i \in H_{\mathbf{p}}} I\{A_i(\mathbf{p}, \delta)\}$ is equal to 0 if the nearest neighbours in $H_{\mathbf{p}}$ are in the δ-ball of $\mathbf{p}$ and is equal to 1 if they are outside, ie, it coincides with $I\{A(\mathbf{p}, \delta)\}$. Therefore, (A.2) can be rewritten as

$$
|\hat{f}(\mathbf{p}) - f(\mathbf{p})| \leq LI\{A(\mathbf{p}, \delta)\} + \Delta(\mathbf{p}, \delta). \tag{A.3}
$$

Taking expectations of both sizes of (A.3), result (6) immediately follows. Moreover, from (A.3), it follows that

$$
\begin{aligned}
||\hat{f} - f||_\infty = \text{sup}_{\mathbf{p} \in D} |\hat{f}(\mathbf{p}) - f(\mathbf{p})| &\leq \text{sup}_{\mathbf{p} \in D} \Delta(\mathbf{p}, \delta) + L\text{sup}_{\mathbf{p} \in D} I\{A(\mathbf{p}, \delta)\} \\
&\leq \Delta(\delta) + LI\{\bigcup_{\mathbf{p} \in D} A(\mathbf{p}, \delta)\}
\end{aligned} \tag{A.4}
$$

and, considering expectations of both sides of (A.4), result (7) follows.

Web Appendix B. Proof of Theorem 2

By the introduction of the random variable $Z(\mathbf{p}, \delta) = \sum_{i=1}^{n} I\{A_i^c(\mathbf{p}, \delta)\}$, it is apparent that $\Pr\{A(\mathbf{p}, \delta)\} = \Pr\{Z(\mathbf{p}, \delta) = 0\}$. In turn, from Cantelli's inequality it follows that

$$
\Pr\{Z(\mathbf{p}, \delta) = 0\} \leq \frac{\text{var}\{Z(\mathbf{p}, \delta)\}}{E\{Z(\mathbf{p}, \delta)^2\}} \leq \frac{\text{var}\{Z(\mathbf{p}, \delta)\}}{[E\{Z(\mathbf{p}, \delta)\}]^2} \tag{B.5}
$$

where

$$E\{Z(\mathbf{p},\delta)\} = \sum_{i=1}^{n} E\{I\{A_i^c(\mathbf{p},\delta)\}\} = \sum_{i=1}^{n} \Pr\{I\{A_i^c(\mathbf{p},\delta)\}\}$$

and

$$\begin{aligned}
\operatorname{var}\{Z(\mathbf{p},\delta)\} &= \operatorname{var}\{\sum_{i=1}^{n} I\{A_i^c(\mathbf{p},\delta)\}\} = \sum_{i=1}^{n} \operatorname{var}\{I\{A_i^c(\mathbf{p},\delta)\}\} + \sum_{h\neq i=1}^{n} \operatorname{cov}\{I\{A_i^c(\mathbf{p},\delta)\}, I\{A_h^c(\mathbf{p},\delta)\}\} \\
&= \sum_{i=1}^{n} \Pr\{A_i^c(\mathbf{p},\delta)\}[1 - \Pr\{A_i^c(\mathbf{p},\delta)\}] \\
&+ \sum_{h\neq i=1}^{n} [\Pr\{A_i^c(\mathbf{p},\delta) \cap A_h^c(\mathbf{p},\delta)\} - \Pr\{A_i^c(\mathbf{p},\delta)\}\Pr\{A_h^c(\mathbf{p},\delta)\}] \\
&\leq \sum_{i=1}^{n} \Pr\{A_i^c(\mathbf{p},\delta)\} + \sum_{h\neq i=1}^{n} [\frac{\Pr\{A_i^c(\mathbf{p},\delta) \cap A_h^c(\mathbf{p},\delta)\}}{\Pr\{A_i^c(\mathbf{p},\delta)\}\Pr\{A_h^c(\mathbf{p},\delta)\}} - 1]\Pr\{A_i^c(\mathbf{p},\delta)\}\Pr\{A_h^c(\mathbf{p},\delta)\} \\
&\leq \sum_{i=1}^{n} \Pr\{A_i^c(\mathbf{p},\delta)\} + \sum_{h\neq i=1}^{n} [\frac{\Pr\{A_i^c(\mathbf{p},\delta) \cap A_h^c(\mathbf{p},\delta)\}}{\Pr\{A_i^c(\mathbf{p},\delta)\}\Pr\{A_h^c(\mathbf{p},\delta)\}} - 1]^+\Pr\{A_i^c(\mathbf{p},\delta)\}\Pr\{A_h^c(\mathbf{p},\delta)\} \\
&\leq \sum_{i=1}^{n} \Pr\{A_i^c(\mathbf{p},\delta)\} \\
&+ \max_{h\neq i=1,\ldots,n} [\frac{\Pr\{A_i^c(\mathbf{p},\delta) \cap A_h^c(\mathbf{p},\delta)\}}{\Pr\{A_i^c(\mathbf{p},\delta)\}\Pr\{A_h^c(\mathbf{p},\delta)\}} - 1]^+ \sum_{h\neq i=1}^{n} \Pr\{A_i^c(\mathbf{p},\delta)\}\Pr\{A_h^c(\mathbf{p},\delta)\}
\end{aligned}$$

Because

$$[\sum_{i=1}^{n} \Pr\{A_i^c(\mathbf{p},\delta)\}]^2 = \sum_{i=1}^{n} \Pr\{A_i^c(\mathbf{p},\delta)\}^2 + \sum_{h\neq i=1}^{n} \Pr\{A_i^c(\mathbf{p},\delta)\}\Pr\{A_h^c(\mathbf{p},\delta)\}$$

then

$$[\sum_{i=1}^{n} \Pr\{A_i^c(\mathbf{p},\delta)\}]^2 \geq \sum_{h\neq i=1}^{n} \Pr\{A_i^c(\mathbf{p},\delta)\}\Pr\{A_h^c(\mathbf{p},\delta)\}$$

from which

$$\operatorname{var}\{Z(\mathbf{p},\delta)\} \leq \sum_{i=1}^{n} \Pr\{A_i^c(\mathbf{p},\delta)\} + \max_{h\neq i=1,\ldots,n} [\frac{\Pr\{A_i^c(\mathbf{p},\delta) \cap A_h^c(\mathbf{p},\delta)\}}{\Pr\{A_i^c(\mathbf{p},\delta)\}\Pr\{A_h^c(\mathbf{p},\delta)\}} - 1]^+[\sum_{i=1}^{n} \Pr\{A_i^c(\mathbf{p},\delta)\}]^2$$

Therefore, from (B.5) it follows that

$$\Pr\{Z(\mathbf{p},\delta)=0\} \leq \frac{\sum_{i=1}^{n}\Pr\{A_i^c(\mathbf{p},\delta)\} + \max\limits_{h\neq i=1,\ldots,n}[\frac{\Pr\{A_i^c(\mathbf{p},\delta)\cap A_h^c(\mathbf{p},\delta)\}}{\Pr\{A_i^c(\mathbf{p},\delta)\}\Pr\{A_h^c(\mathbf{p},\delta)\}}-1]^{+}[\sum_{i=1}^{n}\Pr\{A_i^c(\mathbf{p},\delta)\}]^2}{[\sum_{i=1}^{n}\Pr\{A_i^c(\mathbf{p},\delta)\}]^2}$$
$$= \frac{1}{\sum_{i=1}^{n}\Pr\{A_i^c(\mathbf{p},\delta)\}} + \max_{h\neq i=1,\ldots,n}[\frac{\Pr\{A_i^c(\mathbf{p},\delta)\cap A_h^c(\mathbf{p},\delta)\}}{\Pr\{A_i^c(\mathbf{p},\delta)\}\Pr\{A_h^c(\mathbf{p},\delta)\}}-1]^{+} \tag{B.6}$$

Web Appendix C. Proof of pointwise consistency under URS

If n locations are randomly and independently selected onto B, then

$$\Pr\{A_i^c(\mathbf{p},\delta)\} = \frac{\pi\delta^2}{\lambda(B)}$$

when $\mathbf{p}$ is an inner point of B, ie, its minimum distance from the edge of B is greater than δ. On the other hand, when $\mathbf{p}$ is an edge point, ie, with minimum distance to the edge of B smaller than δ, then

$$\Pr\{A_i^c(\mathbf{p},\delta)\} < \frac{\pi\delta^2}{\lambda(B)}$$

(see Web Figure 1).

We here suppose that the edge of B is sufficiently regular to ensure that there exists a real $r \in (0,1)$ such that it invariably holds

$$\Pr\{A_i^c(\mathbf{p},\delta)\} \geq r\frac{\pi\delta^2}{\lambda(B)}\ ,\ i=1,\ldots n. \tag{C.7}$$

For example $r = 0.25$ when B is a rectangle (see Web Figure 2).

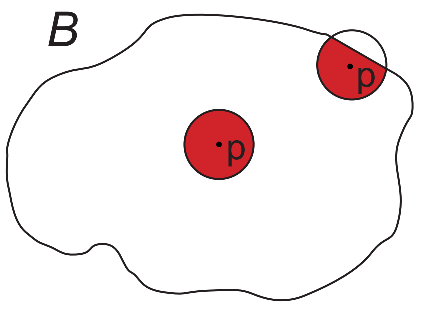

Web Figure 1: Graphical representation of $\Pr\{A_i^c(\mathbf{p}, \delta)\}$ as the ratio of the size of the red area to $\lambda(B)$ for an inner and an edge point.

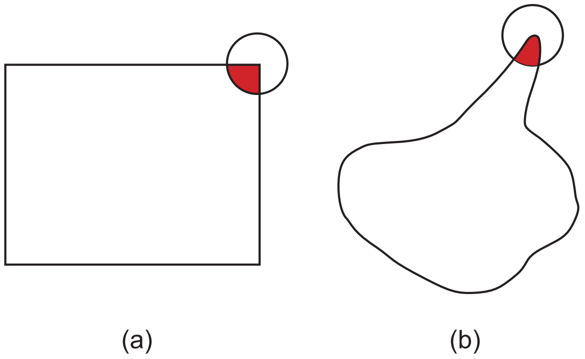

Web Figure 2: Graphical representation of r as the ratio of the size of the red area to the size of the whole ball for two regions with straight (a) and curved (b) edges.

Under condition (C.7), taking in mind that owing to the independence of selections $\Pr\{A_i^c(\mathbf{p},\delta)\cap A_h^c(\mathbf{p},\delta)\} = \Pr\{A_i^c(\mathbf{p},\delta)\}\Pr\{A_h^c(\mathbf{p},\delta)\}$ for each $h \neq i = 1,\ldots,n$, the second term in the right hand side of (B.6) is 0. Then, from (B.6) and (C.7), it follows that

$$\Pr\{A(\mathbf{p},\delta)\} \leq \frac{1}{\sum_{i=1}^{n}\Pr\{A_i^c(\mathbf{p},\delta)\}} \leq \frac{\lambda(B)}{nr\pi\delta^2}. \tag{C.8}$$

Therefore, taking $\delta_k = tn_k^{-1/2}$ for any $t > 0$, from (C.8) it follows that

$$\Pr\{A(\mathbf{p}, tn_k^{-1/2})\} \leq \frac{\lambda(B)}{r\pi t^2}.$$

Accordingly, for any arbitrary $\varepsilon > 0$ taking $t > \sqrt{\frac{\lambda(B)}{r\pi\varepsilon}}$, condition (12) invariably holds for any integer k, proving the pointwise consistency under URS at any continuity point of f_k.

Web Appendix D. Proof of pointwise consistency under 3P sampling

When dealing with the consistency of (1) under 3P sampling it should be considered that 3P sampling, as any other Poisson sampling, is a scheme selecting samples of random size. Therefore Theorem 2 cannot be exploited for the proof as it requires n fixed. Fortunately, stated the simplicity of the scheme, we can work directly with the probability of the event A, without resorting to the A_is. Under 3P sampling, for each $\mathbf{p}_j \in B$, $A(\mathbf{p}_j,\delta)$ is the event that no sample unit lies in the δ-ball of $\mathbf{p}_j$, say $B_j(\delta)$. Therefore, owing to the independence of drawings, for each $\mathbf{p}_j \in B$ it holds that

$$\Pr\{A(\mathbf{p}_j,\delta)\} = \prod_{i\in B_j(\delta)\cap B}(1-\frac{x_i}{L^*}) \leq (1-\pi_0)^{\mathrm{Card}[B_j(\delta)\cap B]}. \tag{D.9}$$

However, owing to the regularity condition (14), for any natural number m there exist a real number $t > 0$ and an integer k_0 such that $\mathrm{Card}\{B_j(tN_k^{-1/2}) \cap B_k\} > m$ for each $k > k_0$. Therefore, from (D.9) it follows that

$$\Pr\{A(\mathbf{p}_j, tN_k^{-1/2})\} \leq (1 - \pi_0)^{\mathrm{Card}\{B_j(tN_k^{-1/2}) \cap B_k\}} \leq (1 - \pi_0)^m. \tag{D.10}$$

Accordingly, for any arbitrary $\varepsilon > 0$ we can choose m sufficiently large to ensure $(1 - \pi_0)^m < \varepsilon$, in such a way that (D.10) coincides with the pointwise consistency condition (12) with N_k instead of n_k.

Web Appendix E. Proof of Theorem 3

Denote by

$$m\hat{s}e_M^*(\mathbf{p}) = \frac{1}{M} \sum_{m=1}^{M} \{\hat{f}_m^*(\mathbf{p}) - \hat{f}(\mathbf{p})\}^2$$

the bootstrap estimator of $E[\{\hat{f}(\mathbf{p}) - f(\mathbf{p})\}^2]$ in such a way that

$$rm\hat{s}e_M^*(\mathbf{p}) = \{m\hat{s}e_M^*(\mathbf{p})\}^{1/2}$$

is the estimator (15) of the root mean squared error. Since $(\mathbf{P}_{1,m}^*, \ldots \mathbf{P}_{n,m}^*)$ for $m = 1, \ldots, M$ are independent and identically distributed random vectors, owing to the strong law of large numbers, conditional on $\mathbf{P}_1, \ldots, \mathbf{P}_n$, as M increases, $m\hat{s}e_M^*(\mathbf{p})$ converges almost surely to

$$mse^*(\mathbf{p}, \mathbf{P}_1, \ldots, \mathbf{P}_n) = E^*\Big(\frac{I(Q_{\mathbf{p},1}^{*c})}{\mathrm{Card}^2(H_{\mathbf{p},1}^*)}\big[\sum_{i \in H_{\mathbf{p},1}^*} \{\hat{f}(\mathbf{P}_{i,1}^*) - \hat{f}(\mathbf{p})\}\big]^2 \,\Big|\, \mathbf{P}_1, \ldots, \mathbf{P}_n\Big) \tag{E.11}$$

where E^* denotes expectation with respect to the first bootstrap resampling conditional on the sample from which resampling is performed.

Now consider the ratio

$$\hat{r}^*(\mathbf{p}) = \frac{\hat{mse}^*_M(\mathbf{p})}{E[\{\hat{f}(\mathbf{p}) - f(\mathbf{p})\}^2]}$$
$$= \frac{\hat{mse}^*_M(\mathbf{p}) - mse^*(\mathbf{p}, \mathbf{P}_1, \ldots, \mathbf{P}_n)}{E[\{\hat{f}(\mathbf{p}) - f(\mathbf{p})\}^2]} + \frac{mse^*(\mathbf{p}, \mathbf{P}_1, \ldots, \mathbf{P}_n)}{E[\{\hat{f}(\mathbf{p}) - f(\mathbf{p})\}^2]}$$

that, for M sufficiently large, is equivalent to

$$r^*(\mathbf{p}, \mathbf{P}_1, \ldots, \mathbf{P}_n) = \frac{mse^*(\mathbf{p}, \mathbf{P}_1, \ldots, \mathbf{P}_n)}{E[\{\hat{f}(\mathbf{p}) - f(\mathbf{p})\}^2]} \tag{E.12}$$

since $E[\{\hat{mse}^*_M(\mathbf{p}) - mse^*(\mathbf{p}, \mathbf{P}_1, \ldots, \mathbf{P}_n)\}^2] = O(M^{-1})$. In the case of continuous populations and finite populations of units, for $i \in H^*_{\mathbf{p},1}$ it holds that

$$I(Q^{*c}_{\mathbf{p},1})\{\hat{f}(\mathbf{P}^*_{i,1}) - \hat{f}(\mathbf{p})\} = I(Q^{*c}_{\mathbf{p},1})\{\frac{1}{\mathrm{Card}(H^*_{\mathbf{P}^*_{i,1}})} \sum_{l \in H^*_{\mathbf{P}^*_{i,1}}} f(\mathbf{P}_l) - \frac{1}{\mathrm{Card}(H_{\mathbf{p}})} \sum_{l \in H_{\mathbf{p}}} f(\mathbf{P}_l)\} \tag{E.13}$$

where $H^*_{\mathbf{P}^*_{i,1}} = \{l : \ \|\mathbf{P}_l - \mathbf{P}^*_{i,1}\| = \min_{h=1,\ldots n}\|\mathbf{P}_h - \mathbf{P}^*_{i,1}\|\}$. Then, owing to condition (18), equation (E.13) reduces to

$$I(Q^{*c}_{\mathbf{p},1})\{\hat{f}(\mathbf{P}^*_{i,1}) - \hat{f}(\mathbf{p})\} = I(Q^{*c}_{\mathbf{p},1})(\langle \mathbf{a}, \mathbf{X}_{i,1}\rangle + g_{i,1})$$

where

$$\mathbf{X}_{i,1} = \frac{1}{\mathrm{Card}(H^*_{\mathbf{P}^*_{i,1}})} \sum_{l \in H^*_{\mathbf{P}^*_{i,1}}} (\mathbf{P}_l - \mathbf{p}) - \frac{1}{\mathrm{Card}(H_{\mathbf{p}})} \sum_{l \in H_{\mathbf{p}}} (\mathbf{P}_l - \mathbf{p})$$

and

$$g_{i,1} = \frac{1}{\mathrm{Card}(H^*_{\mathbf{P}^*_{i,1}})} \sum_{l \in H^*_{\mathbf{P}^*_{i,1}}} o\left(\|\mathbf{P}_l - \mathbf{p}\|\right) - \frac{1}{\mathrm{Card}(H_{\mathbf{p}})} \sum_{l \in H_{\mathbf{p}}} o\left(\|\mathbf{P}_l - \mathbf{p}\|\right).$$

Therefore, the quantity in equation (E.11) can be rewritten as

$$\frac{I(Q^{*c}_{\mathbf{p},1})}{\mathrm{Card}^2(H^*_{\mathbf{p},1})}\Big[\sum_{i \in H^*_{\mathbf{p},1}} \{\hat{f}(\mathbf{P}^*_{i,1}) - \hat{f}(\mathbf{p})\}\Big]^2 = \frac{I(Q^{*c}_{\mathbf{p},1})}{\mathrm{Card}^2(H^*_{\mathbf{p},1})}(\langle \mathbf{a}, \mathbf{X}_{\mathbf{p},1}\rangle - \sum_{i \in H^*_{\mathbf{p},1}} g_{i,1})^2 \tag{E.14}$$

where $\mathbf{X}_\mathbf{p} = \sum_{i \in H^*_{\mathbf{p},1}} \mathbf{X}_{i,1}$. The same result can be achieved in the case of finite populations of areas, integrating f over the areas. Accordingly, for all the kinds of populations, if δ is sufficiently small, equation (E.14) is equivalent to

$$\frac{I(Q^{*c}_{\mathbf{p},1})}{\mathrm{Card}^2(H^*_{\mathbf{p},1})}\Big[\sum_{i \in H^*_{\mathbf{p},1}} \{\hat{f}(\mathbf{P}^*_{i,1}) - \hat{f}(\mathbf{p})\}\Big]^2 = \frac{I(Q^{*c}_{\mathbf{p},1})}{\mathrm{Card}^2(H^*_{\mathbf{p},1})}\langle \mathbf{a}, \mathbf{X}_\mathbf{p}\rangle^2$$

from which the ratio (E.12) is equivalent to

$$\begin{aligned} r^*(\mathbf{p}, \mathbf{P}_1, \ldots, \mathbf{P}_n) &= \frac{E^*\{\frac{I(Q^{*c}_{\mathbf{p},1})}{\mathrm{Card}^2(H^*_{\mathbf{p},1})}\langle \mathbf{a}, \mathbf{X}_\mathbf{p}\rangle^2 | \mathbf{P}_1, \ldots, \mathbf{P}_n\}}{E[\{\hat{f}(\mathbf{p}) - f(\mathbf{p})\}^2]} \\ &= \frac{E^*\{\frac{I(Q^{*c}_{\mathbf{p},1})}{\mathrm{Card}^2(H^*_{\mathbf{p},1})}\langle \mathbf{a}, \mathbf{X}_\mathbf{p}\rangle^2 | \mathbf{P}_1, \ldots, \mathbf{P}_n\}}{E[\frac{1}{\mathrm{Card}^2(H_\mathbf{p})}\{\langle \mathbf{a}, \sum_{i \in H_\mathbf{p}} (\mathbf{P}_i - \mathbf{p})\rangle\}^2]}. \end{aligned} \tag{E.15}$$

Adding and subtracting $\frac{1}{\mathrm{Card}(H^*_{\mathbf{p},1})} \sum_{i \in H^*_{\mathbf{p},1}} (\mathbf{P}^*_{i,1} - \mathbf{p})$ to $\mathbf{X}_\mathbf{p}$ into equation (E.15) the following inequality holds

$$\begin{aligned} r^*(\mathbf{p}, \mathbf{P}_1, \ldots, \mathbf{P}_n) \leq 3\Big(1 &+ \frac{\frac{I(Q^c_\mathbf{p})}{\mathrm{Card}^2(H_\mathbf{p})}\{\langle \mathbf{a}, \sum_{i \in H_\mathbf{p}} (\mathbf{P}_i - \mathbf{p})\rangle\}^2}{E[\frac{1}{\mathrm{Card}^2(H_\mathbf{p})}\{\langle \mathbf{a}, \sum_{i \in H_\mathbf{p}} (\mathbf{P}_i - \mathbf{p})\rangle\}^2]} \\ &+ \frac{E^*\{\frac{I(Q^{*c}_{\mathbf{p},1})}{\mathrm{Card}^2(H^*_{\mathbf{p},1})}\langle \mathbf{a}, \mathbf{Z}_\mathbf{p}\rangle^2 | \mathbf{P}_1, \ldots, \mathbf{P}_n\}}{E[\frac{1}{\mathrm{Card}^2(H_\mathbf{p})}\{\langle \mathbf{a}, \sum_{i \in H_\mathbf{p}} (\mathbf{P}_i - \mathbf{p})\rangle\}^2]}\Big) \end{aligned} \tag{E.16}$$

where

$$\mathbf{Z}_\mathbf{p} = \sum_{i \in H^*_{\mathbf{p},1}} \frac{1}{\mathrm{Card}(H^*_{\mathbf{p},1})} \sum_{l \in H^*_{\mathbf{P}^*_{i,1}}} (\mathbf{P}_l - \mathbf{P}^*_{i,1}).$$

Taking the expectation with respect to $\mathbf{P}_1, \ldots, \mathbf{P}_n$ of both sides of (E.16), the expectation of the third term of the sum can be bounded by 1, in such a way that $E\{r^*(\mathbf{p}, \mathbf{P}_1, \ldots, \mathbf{P}_n)\} \leq 9$. Therefore

$$E\{\sqrt{\hat{r}^*(\mathbf{p})}\} \leq E\{\hat{r}^*(\mathbf{p})\}^{1/2} \approx E\{r^*(\mathbf{p}, \mathbf{P}_1, \ldots, \mathbf{P}_n)\}^{1/2} \leq 3.$$

Web Appendix F. Tables and maps from simulation study

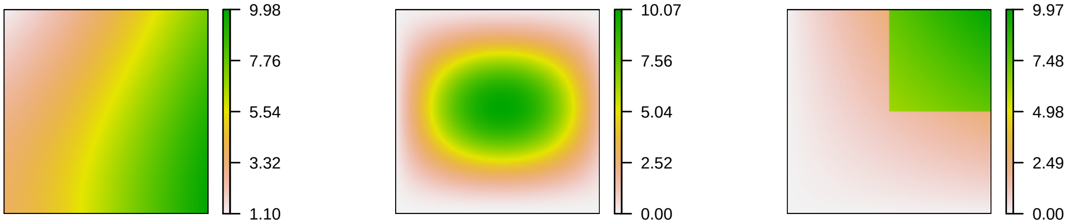

Web Figure 3: Maps of the three artificial surfaces.

Web Table 1: Mimima, means and maxima of absolute bias (AB), root mean squared error (RMSE) and bootsrap ratio (BORAT) for continuous population generated from surface 1

Scheme	n	AB			RMSE			BORAT		
		min	mean	max	min	mean	max	min	mean	max
URS	16	0.00	0.17	1.24	0.52	0.77	1.51	0.55	1.28	1.52
	36	0.00	0.08	0.79	0.32	0.50	0.98	0.55	1.36	1.52
	64	0.00	0.04	0.58	0.22	0.37	0.72	0.54	1.40	1.53
	100	0.00	0.03	0.44	0.17	0.29	0.56	0.56	1.42	1.53
TSS	16	0.00	0.15	0.95	0.34	0.60	1.09	0.42	1.33	1.59
	36	0.00	0.08	0.60	0.21	0.40	0.69	0.41	1.40	1.60
	64	0.00	0.06	0.43	0.15	0.29	0.50	0.41	1.43	1.60
	100	0.00	0.04	0.33	0.12	0.23	0.39	0.41	1.45	1.59
SGS	16	0.00	0.13	0.96	0.34	0.55	1.09	0.43	1.38	1.67
	36	0.00	0.06	0.60	0.21	0.35	0.70	0.43	1.48	1.68
	64	0.00	0.03	0.43	0.15	0.26	0.51	0.44	1.52	1.69
	100	0.00	0.02	0.34	0.12	0.21	0.40	0.45	1.55	1.69

Web Table 2: Mimima, means and maxima of absolute bias (AB), root mean squared error (RMSE) and bootsrap ratio (BORAT) for continuous population generated from surface 2

Scheme	n	AB			RMSE			BORAT		
		min	mean	max	min	mean	max	min	mean	max
URS	16	0.00	0.79	2.53	1.60	2.13	3.21	0.73	1.11	1.27
	36	0.00	0.37	1.91	0.75	1.38	2.44	0.74	1.27	1.42
	64	0.00	0.21	1.45	0.37	1.01	1.87	0.77	1.34	1.47
	100	0.00	0.14	1.18	0.20	0.79	1.54	0.79	1.38	1.50
TSS	16	0.00	0.58	2.53	0.69	1.69	3.06	0.59	1.18	1.52
	36	0.00	0.30	1.84	0.23	1.09	2.23	0.60	1.32	1.63
	64	0.00	0.18	1.42	0.10	0.81	1.73	0.60	1.38	1.70
	100	0.00	0.13	1.13	0.05	0.64	1.39	0.62	1.41	1.74
SGS	16	0.01	0.61	3.24	0.68	1.66	3.77	0.42	1.19	1.55
	36	0.00	0.27	2.25	0.24	1.02	2.63	0.42	1.35	1.65
	64	0.00	0.15	1.70	0.11	0.74	2.00	0.43	1.42	1.68
	100	0.00	0.09	1.33	0.06	0.58	1.58	0.44	1.46	1.70

Web Table 3: Mimima, means and maxima of absolute bias (AB), root mean squared error (RMSE) and bootsrap ratio (BORAT) for continuous population generated from surface 3

Scheme	n	AB			RMSE			BORAT		
		min	mean	max	min	mean	max	min	mean	max
URS	16	0.00	0.50	3.66	0.15	1.26	4.42	0.59	1.33	2.02
	36	0.00	0.31	3.61	0.07	0.83	4.34	0.53	1.38	2.56
	64	0.00	0.22	3.52	0.04	0.62	4.26	0.49	1.42	2.93
	100	0.00	0.18	3.46	0.03	0.50	4.21	0.51	1.44	2.88
TSS	16	0.00	0.31	3.59	0.07	0.83	4.37	0.35	1.53	4.68
	36	0.00	0.21	3.55	0.03	0.56	4.30	0.36	1.60	5.58
	64	0.00	0.15	3.49	0.02	0.43	4.24	0.37	1.63	6.70
	100	0.00	0.12	3.32	0.01	0.34	4.13	0.38	1.64	7.66
SGS	16	0.00	0.37	3.61	0.07	0.84	4.36	0.37	1.57	7.52
	36	0.00	0.24	3.57	0.03	0.56	4.30	0.39	1.70	11.07
	64	0.00	0.17	3.52	0.02	0.42	4.25	0.40	1.78	14.43
	100	0.00	0.14	3.49	0.01	0.34	4.22	0.42	1.79	16.63

Web Table 4: Mimima, means and maxima for the values of absolute bias (AB), root mean squared error (RMSE) and bootstrap ratio (BORAT) achieved for populations of quadrats generated from surface 1 with 10% sampling fraction

Scheme	Grid	AB			RMSE			BORAT		
		min	mean	max	min	mean	max	min	mean	max
SRSWOR	10x10	0.00	0.27	1.25	0.67	0.95	1.60	0.63	1.19	1.52
	20x20	0.00	0.08	0.59	0.29	0.45	0.79	0.64	1.35	1.54
	30x30	0.00	0.04	0.39	0.18	0.29	0.52	0.62	1.39	1.53
	40x40	0.00	0.03	0.28	0.12	0.22	0.38	0.63	1.42	1.55
OPSS	10x10	0.00	0.27	1.13	0.59	0.86	1.42	0.55	1.18	1.62
	20x20	0.00	0.08	0.54	0.25	0.40	0.68	0.55	1.35	1.65
	30x30	0.00	0.05	0.36	0.15	0.26	0.46	0.55	1.39	1.63
	40x40	0.00	0.02	0.25	0.11	0.20	0.33	0.53	1.44	1.64
SYS	10x10	0.00	0.45	1.60	0.77	1.10	1.88	0.42	0.95	1.54
	20x20	0.00	0.13	0.79	0.33	0.50	0.93	0.42	1.22	1.55
	30x30	0.00	0.06	0.55	0.20	0.32	0.65	0.43	1.34	1.57
	40x40	0.00	0.04	0.40	0.15	0.24	0.48	0.40	1.37	1.57

Web Table 5: Mimima, means and maxima for the values of absolute bias (AB), root mean squared error (RMSE) and bootstrap ratio (BORAT) achieved for populations of quadrats generated from surface 2 with 10% sampling fraction

Scheme	Grid	AB			RMSE			BORAT		
		min	mean	max	min	mean	max	min	mean	max
SRSWOR	10x10	0.00	1.14	2.28	2.15	2.57	3.18	0.71	0.95	1.10
	20x20	0.00	0.33	1.32	0.62	1.24	1.95	0.85	1.26	1.46
	30x30	0.00	0.15	0.96	0.22	0.80	1.41	0.86	1.34	1.52
	40x40	0.00	0.09	0.73	0.10	0.59	1.06	0.88	1.38	1.52
OPSS	10x10	0.00	0.89	2.09	0.96	2.18	3.03	0.80	1.10	1.51
	20x20	0.00	0.27	1.33	0.15	1.03	1.89	0.79	1.34	1.84
	30x30	0.00	0.14	0.94	0.05	0.67	1.33	0.81	1.39	1.85
	40x40	0.00	0.08	0.70	0.02	0.50	0.99	0.82	1.44	1.94
SYS	10x10	0.13	1.18	4.42	0.63	2.00	5.27	0.25	0.75	1.73
	20x20	0.02	0.51	2.81	0.13	1.02	3.42	0.07	0.90	1.82
	30x30	0.00	0.22	1.93	0.04	0.60	2.37	0.37	1.18	1.87
	40x40	0.00	0.17	1.49	0.02	0.46	1.81	0.02	1.10	2.32

Web Table 6: Mimima, means and maxima for the values of absolute bias (AB), root mean squared error (RMSE) and bootstrap ratio (BORAT) achieved for populations of quadrats generated from surface 3 with 10% sampling fraction

Scheme	Grid	AB			RMSE			BORAT		
		min	mean	max	min	mean	max	min	mean	max
SRSWOR	10x10	0.00	0.61	3.06	0.28	1.53	4.15	0.73	1.24	1.83
	20x20	0.00	0.27	3.04	0.06	0.74	4.01	0.59	1.36	2.36
	30x30	0.00	0.16	2.49	0.03	0.48	3.61	0.56	1.42	2.78
	40x40	0.00	0.15	3.05	0.02	0.40	3.96	0.59	1.45	2.91
OPSS	10x10	0.00	0.44	2.97	0.12	1.12	4.08	0.78	1.43	2.66
	20x20	0.00	0.20	2.88	0.03	0.56	3.90	0.50	1.55	4.50
	30x30	0.00	0.12	2.33	0.01	0.37	3.50	0.51	1.58	7.22
	40x40	0.00	0.12	2.89	0.01	0.31	3.85	0.53	1.70	12.60
SYS	10x10	0.00	0.59	3.65	0.11	1.07	4.46	0.35	1.24	3.05
	20x20	0.00	0.30	3.55	0.03	0.58	4.30	0.16	1.36	10.47
	30x30	0.00	0.14	2.10	0.01	0.31	3.36	0.56	1.42	2.78
	40x40	0.00	0.16	3.54	0.01	0.32	4.25	0.02	1.56	21.29

Web Table 7: Mimima, means and maxima of absolute bias (AB), root mean squared error (RMSE) and bootstrap ratio (BORAT) for nested populations of $500, 1000, 1500$ units having Y-values from 4 to 10 generated from surface 1 under 3P sampling with constant $L^* = 50$

Pattern	N	$E[n]$	AB			RMSE			BORAT		
			min	mean	max	min	mean	max	min	mean	max
Regular	358	45.3	0.00	0.02	0.12	0.05	0.09	0.16	0.69	1.28	1.56
	729	91.6	0.00	0.01	0.09	0.04	0.06	0.12	0.71	1.35	1.55
	1102	138.8	0.00	0.01	0.09	0.04	0.05	0.11	0.77	1.37	1.56
Random	349	43.7	0.00	0.02	0.11	0.05	0.09	0.15	0.70	1.26	1.62
	711	88.7	0.00	0.01	0.08	0.04	0.06	0.11	0.64	1.29	1.60
	1059	131.5	0.00	0.01	0.07	0.03	0.05	0.09	0.64	1.32	1.57
Trended	404	54.7	0.00	0.02	0.17	0.01	0.07	0.22	0.69	1.21	1.58
	807	108.7	0.00	0.01	0.11	0.01	0.05	0.15	0.71	1.25	1.60
	1231	165.8	0.00	0.01	0.08	0.01	0.04	0.12	0.69	1.28	1.62
Clustered	413	53.2	0.00	0.01	0.10	0.01	0.03	0.17	0.41	098	1.40
	827	106.4	0.00	0.01	0.07	0.01	0.02	0.09	0.43	1.12	1.45
	1237	159.4	0.00	0.01	0.06	0.01	0.02	0.07	0.41	1.18	1.52

Web Table 8: Mimima, means and maxima of absolute bias (AB), root mean squared error (RMSE) and bootstrap ratio (BORAT) for nested populations of $500, 1000, 1500$ units having Y-values from 4 to 10 generated from surface 2 under 3P sampling with constant $L^* = 50$

Pattern	N	$E[n]$	AB			RMSE			BORAT		
			min	mean	max	min	mean	max	min	mean	max
Regular	179	23.1	0.00	0.11	0.40	0.08	0.27	0.51	0.63	1.10	1.47
	387	50.8	0.00	0.06	0.29	0.03	0.18	0.38	0.69	1.23	1.53
	579	76.0	0.00	0.04	0.25	0.02	0.15	0.32	0.71	1.27	1.54
Random	179	22.9	0.00	0.11	0.42	0.11	0.27	0.51	0.68	1.07	1.43
	365	47.0	0.00	0.06	0.31	0.05	0.19	0.40	0.58	1.17	1.48
	571	74.2	0.00	0.04	0.28	0.03	0.15	0.35	0.55	1.21	1.50
Trended	105	13.4	0.00	0.16	0.43	0.14	0.35	0.58	0.60	0.98	1.40
	210	26.7	0.00	0.09	0.36	0.06	0.25	0.47	0.62	1.11	1.56
	327	41.7	0.00	0.07	0.34	0.04	0.20	0.43	0.64	1.15	1.57
Clustered	154	18.8	0.00	0.06	0.45	0.03	0.14	0.69	0.50	0.92	1.28
	316	38.5	0.00	0.03	0.17	0.02	0.09	0.35	0.51	1.05	1.46
	473	57.7	0.00	0.02	0.19	0.02	0.06	0.26	0.47	1.11	1.48

Web Table 9: Mimima, means and maxima of absolute bias (AB), root mean squared error (RMSE) and bootstrap ratio (BORAT) for nested populations of $500, 1000, 1500$ units having Y-values from 4 to 10 generated from surface 3 under 3P sampling with constant $L^* = 50$

Pattern	N	$E[n]$	AB			RMSE			BORAT		
			min	mean	max	min	mean	max	min	mean	max
Regular	131	19.1	0.00	0.02	0.10	0.04	0.06	0.13	0.60	1.23	1.54
	260	37.9	0.00	0.01	0.05	0.03	0.04	0.07	0.72	1.31	1.53
	373	54.7	0.00	0.01	0.07	0.02	0.03	0.08	0.48	1.34	1.54
Random	124	18.1	0.00	0.02	0.08	0.04	0.06	0.11	0.60	1.21	1.48
	251	36.7	0.00	0.01	0.07	0.03	0.04	0.08	0.60	1.24	1.53
	366	53.4	0.00	0.01	0.06	0.02	0.03	0.07	0.58	1.26	1.57
Trended	251	39.7	0.00	0.01	0.11	0.01	0.04	0.13	0.49	1.22	1.60
	513	81.1	0.00	0.01	0.07	0.00	0.03	0.09	0.61	1.24	1.57
	783	123.5	0.00	0.01	0.05	0.00	0.02	0.07	0.64	1.26	1.58
Clustered	166	24.9	0.00	0.01	0.06	0.01	0.02	0.08	0.38	1.00	1.39
	339	50.7	0.00	0.01	0.04	0.01	0.01	0.05	0.40	1.12	1.46
	511	76.3	0.00	0.00	0.04	0.00	0.01	0.04	0.44	1.18	1.53

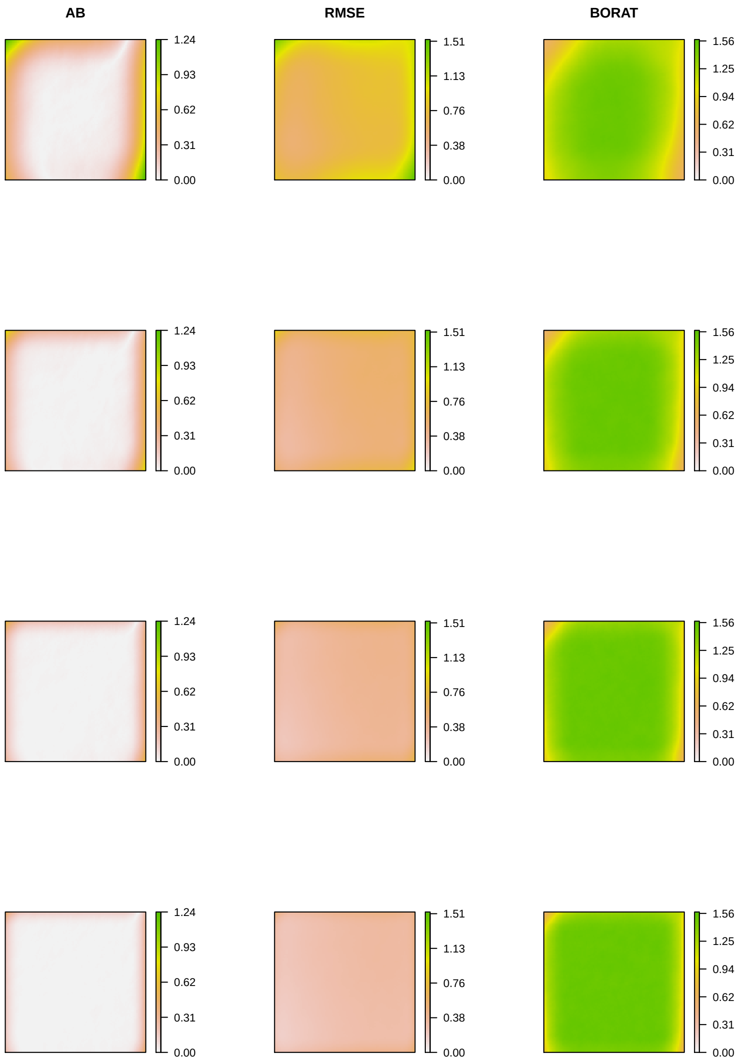

Web Figure 4: Maps of absolute bias (AB), root mean squared error (RMSE) and bootstrap ratio (BORAT) for continuous population from surface 1 under URS of $n = 16, 36, 64, 100$ locations (rows 1-4 respectively).

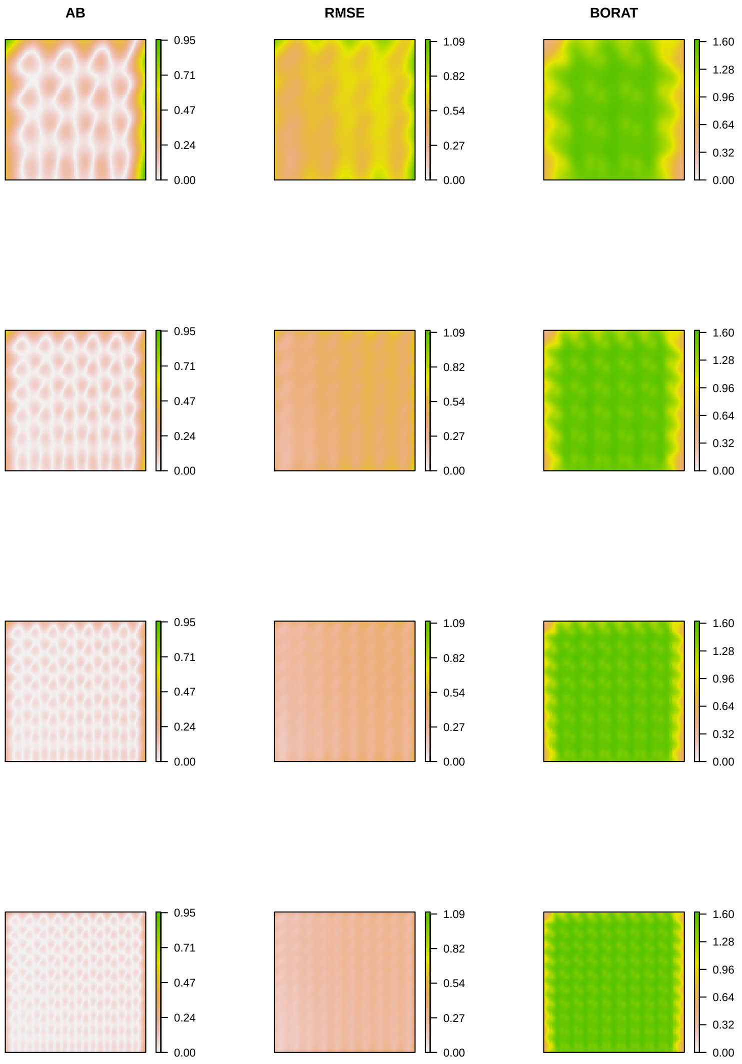

Web Figure 5: Maps of absolute bias (AB), root mean squared error (RMSE) and bootstrap ratio (BORAT) for continuous population from surface 1 under TSS of $n = 16, 36, 64, 100$ locations (rows 1-4 respectively).

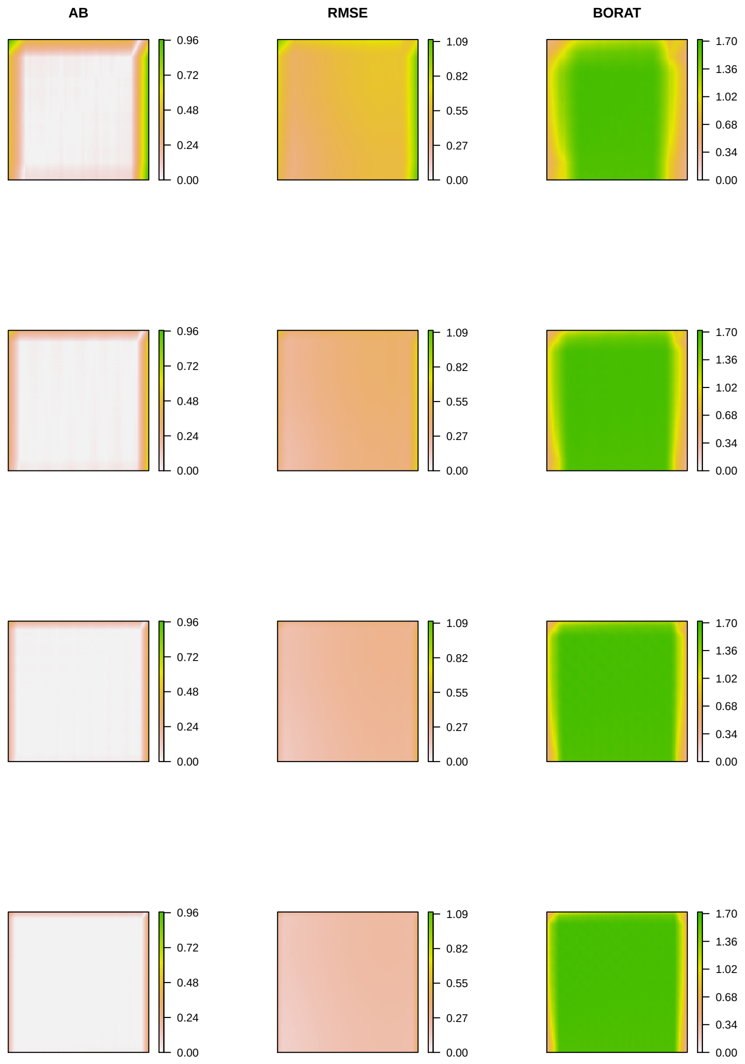

Web Figure 6: Maps of absolute bias (AB), root mean squared error (RMSE) and bootstrap ratio (BORAT) for continuous population from surface 1 under SGS of $n = 16, 36, 64, 100$ locations (rows 1-4 respectively).

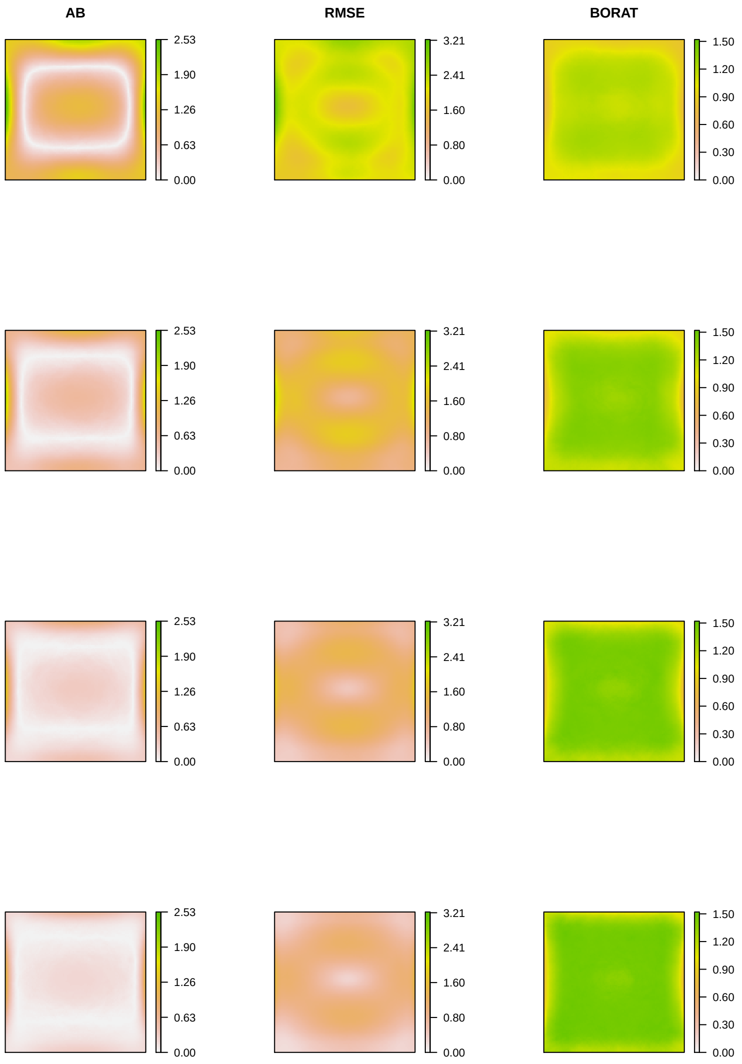

Web Figure 7: Maps of absolute bias (AB), root mean squared error (RMSE) and bootstrap ratio (BORAT) for continuous population from surface 2 under URS of $n = 16, 36, 64, 100$ locations (rows 1-4 respectively).

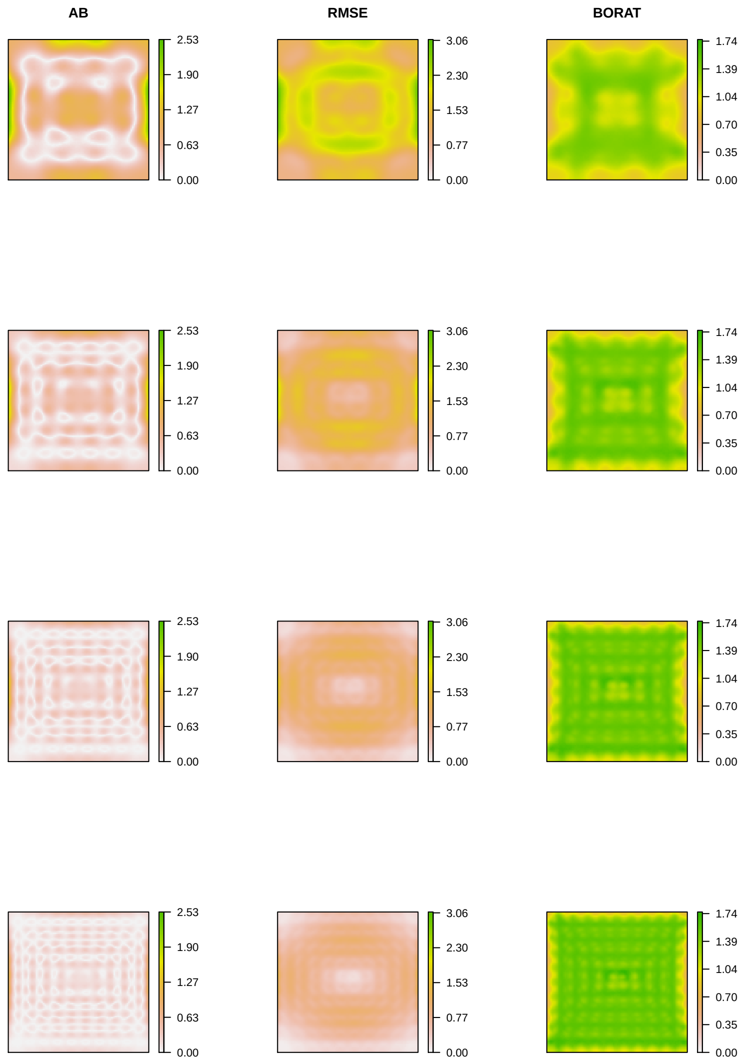

Web Figure 8: Maps of absolute bias (AB), root mean squared error (RMSE) and bootstrap ratio (BORAT) for continuous population from surface 2 under TSS of $n = 16, 36, 64, 100$ locations (rows 1-4 respectively).

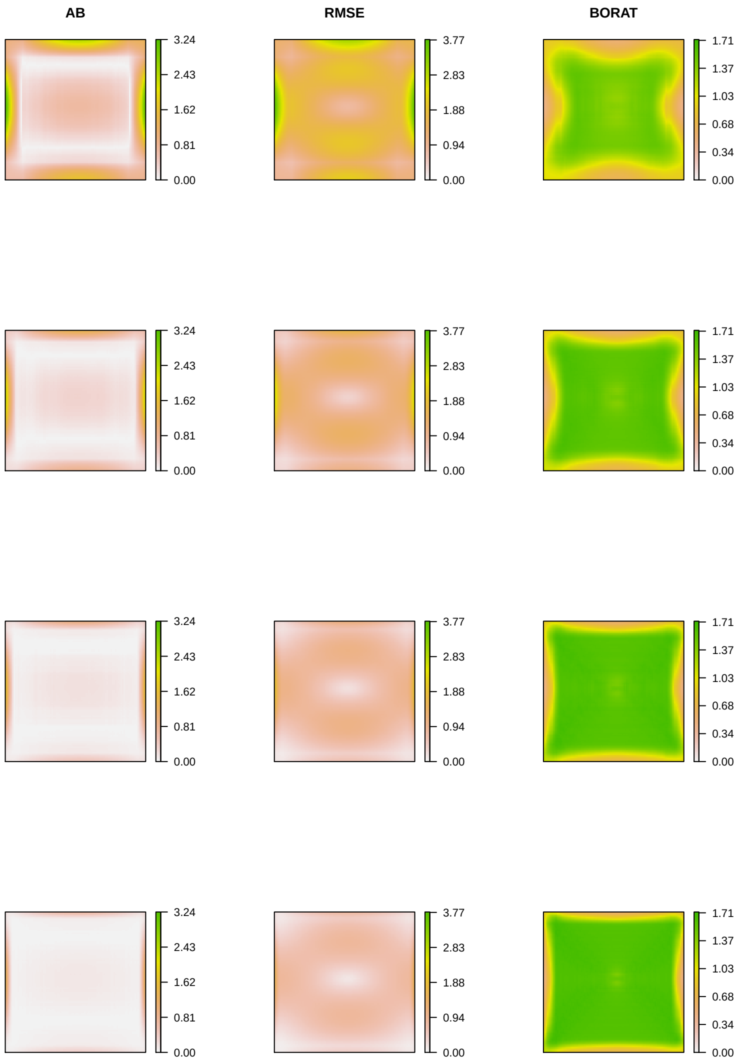

Web Figure 9: Maps of absolute bias (AB), root mean squared error (RMSE) and bootstrap ratio (BORAT) for continuous population from surface 2 under SGS of $n = 16, 36, 64, 100$ locations (rows 1-4 respectively).

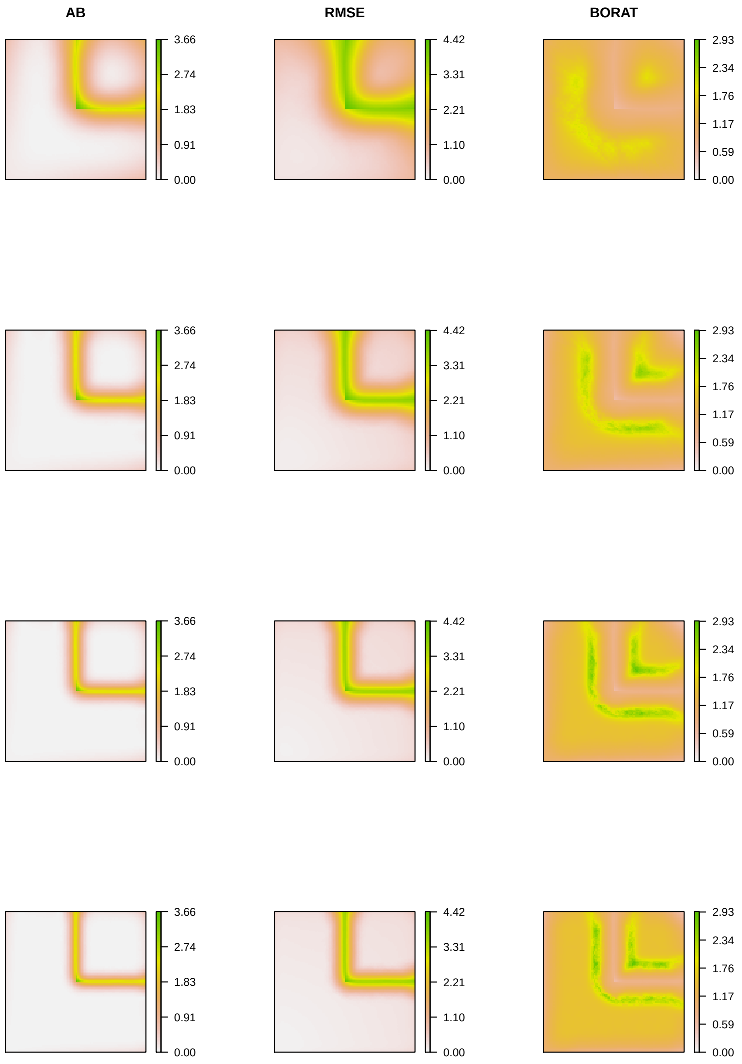

Web Figure 10: Maps of absolute bias (AB), root mean squared error (RMSE) and bootstrap ratio (BORAT) for continuous population from surface 3 under URS of $n = 16, 36, 64, 100$ locations (rows 1-4 respectively).

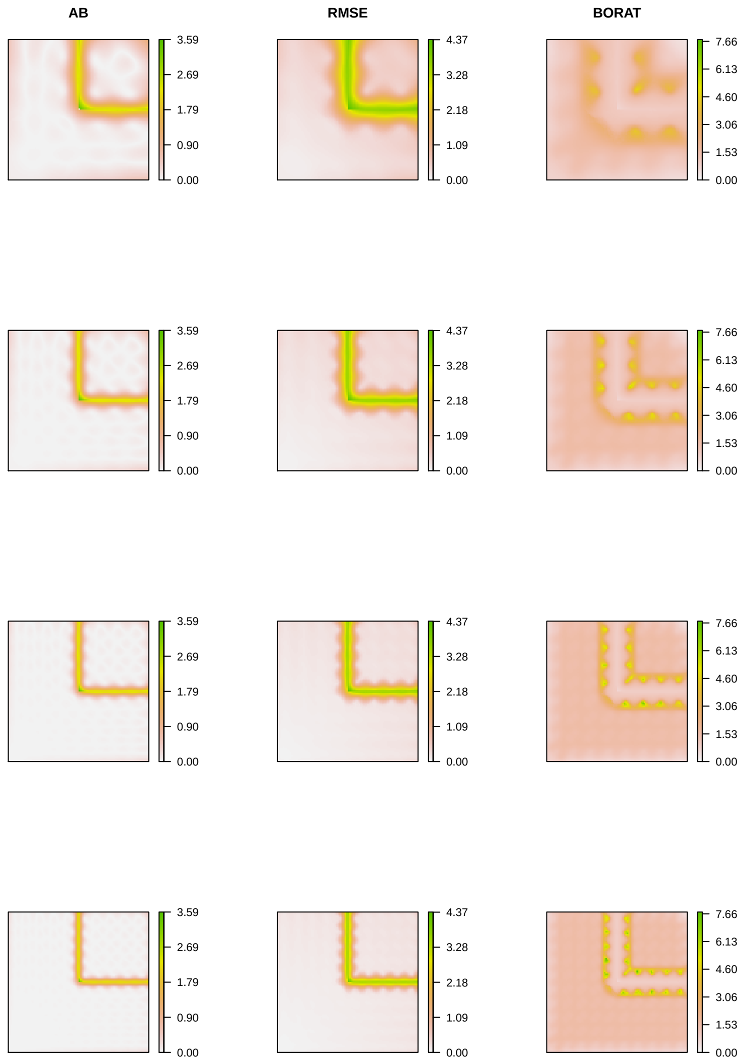

Web Figure 11: Maps of absolute bias (AB), root mean squared error (RMSE) and bootstrap ratio (BORAT) for continuous population from surface 3 under TSS of $n = 16, 36, 64, 100$ locations (rows 1-4 respectively)

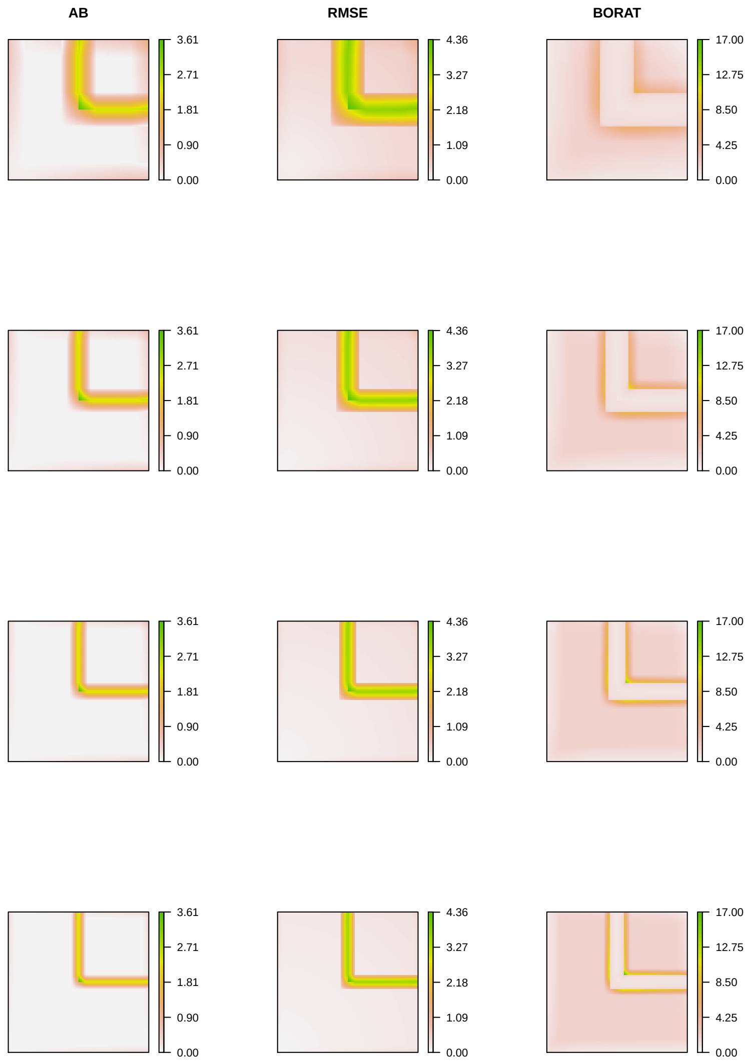

Web Figure 12: Maps of absolute bias (AB), root mean squared error (RMSE) and bootstrap ratio (BORAT) for continuous population from surface 3 under SGS of $n = 16, 36, 64, 100$ locations (rows 1-4 respectively).

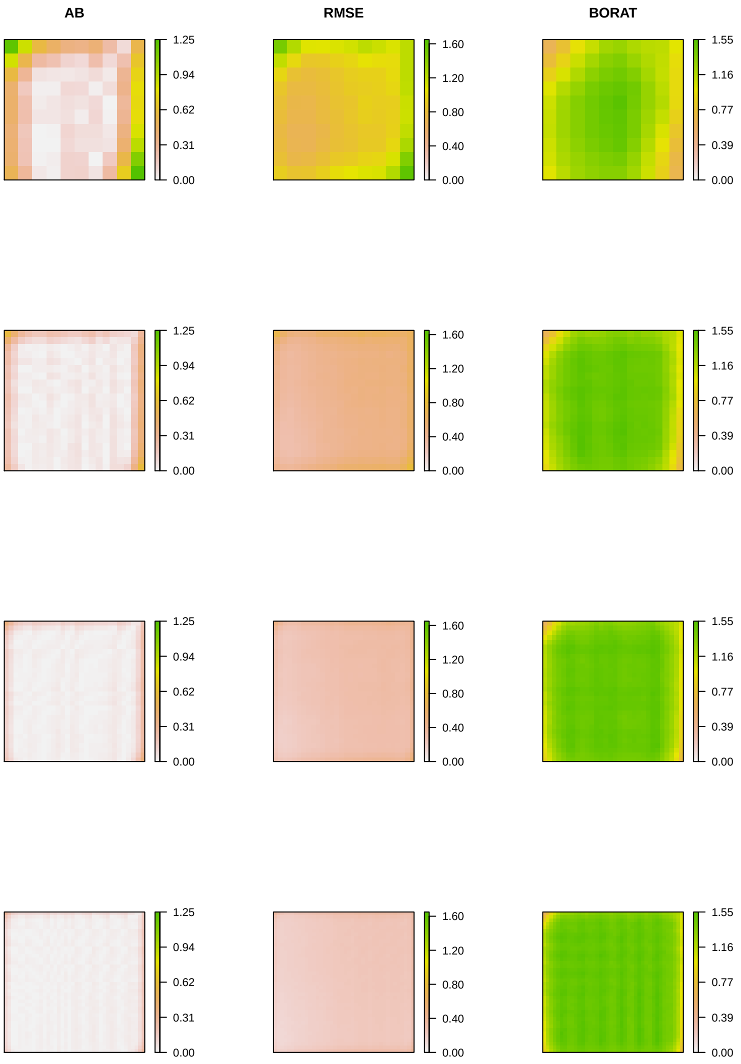

Web Figure 13: Maps of absolute bias (AB), root mean squared error (RMSE) and bootstrap ratio (BORAT) for populations of 100, 400, 900, 1600 quadrats from surface 1 under SRSWOR with 10% sampling fraction (rows 1-4 respectively).

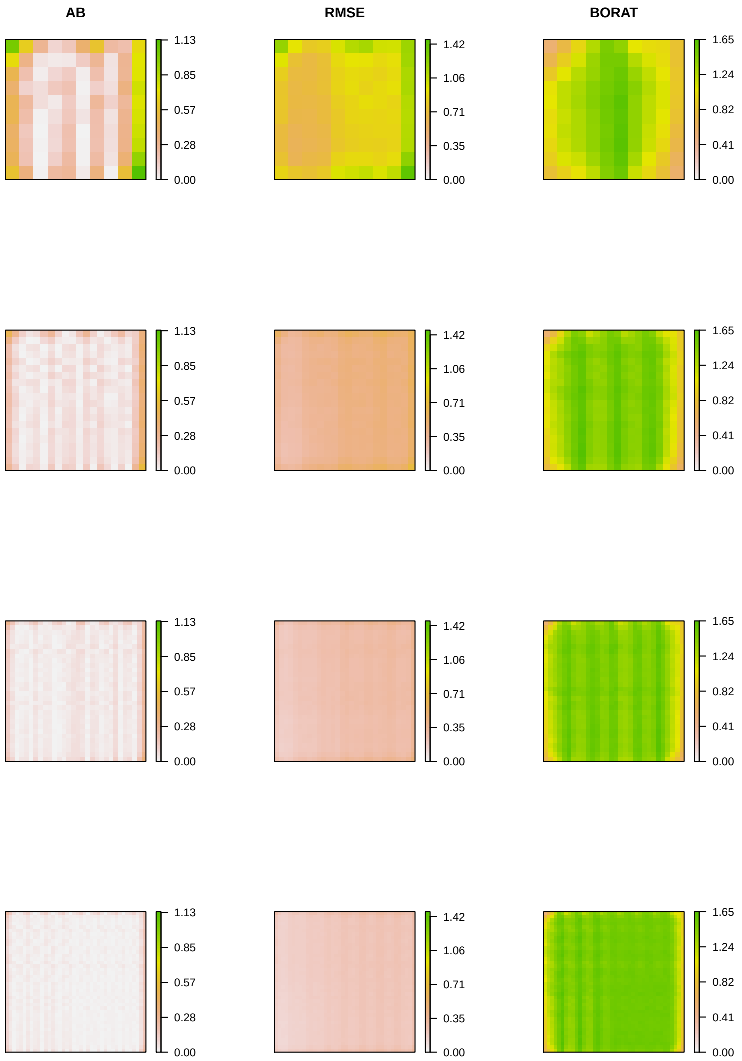

Web Figure 14: Maps of absolute bias (AB), root mean squared error (RMSE) and bootstrap ratio (BORAT) for populations of 100, 400, 900, 1600 quadrats from surface 1 under OPSS with 10% sampling fraction (rows 1-4 respectively).

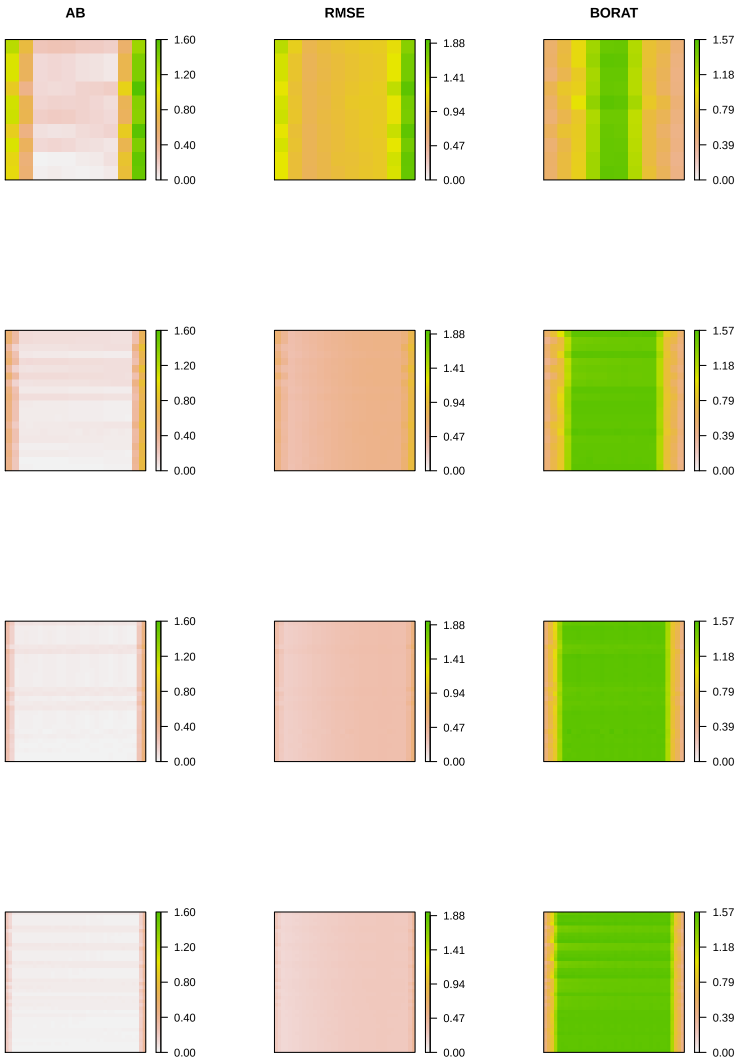

Web Figure 15: Maps of absolute bias (AB), root mean squared error (RMSE) and bootstrap ratio (BORAT) for populations of 100, 400, 900, 1600 quadrats from surface 1 under SYS with 10% sampling fraction (rows 1-4 respectively).

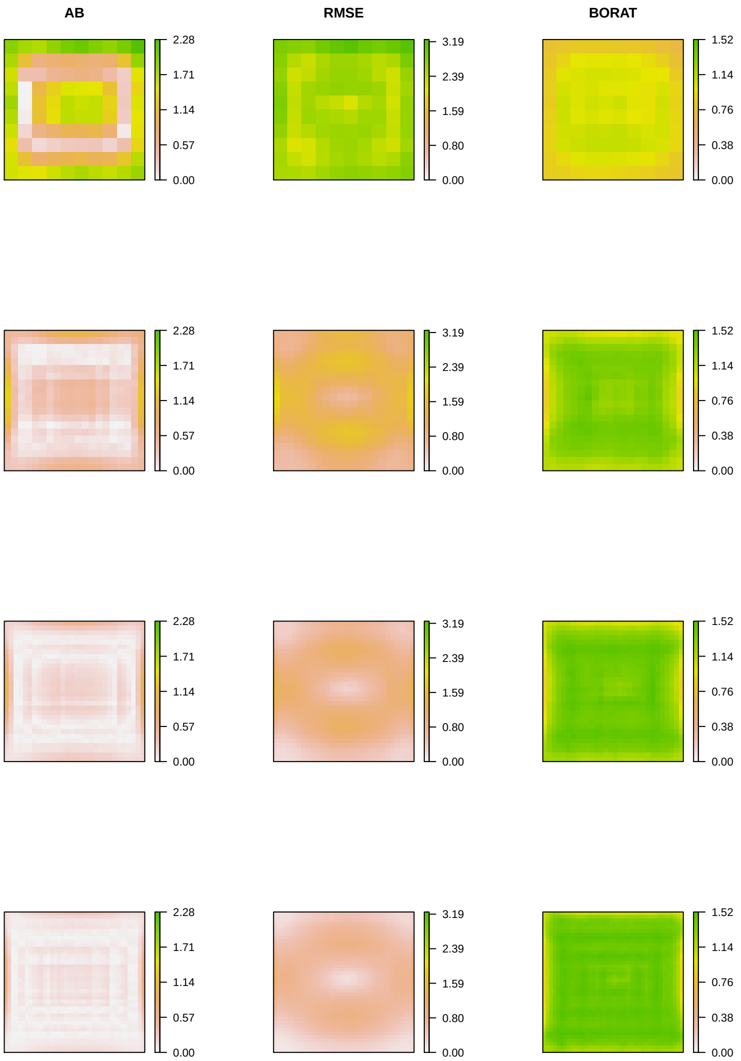

Web Figure 16: Maps of absolute bias (AB), root mean squared error (RMSE) and bootstrap ratio (BORAT) for populations of 100, 400, 900, 1600 quadrats from surface 2 under SRSWOR with 10% sampling fraction (rows 1-4 respectively).

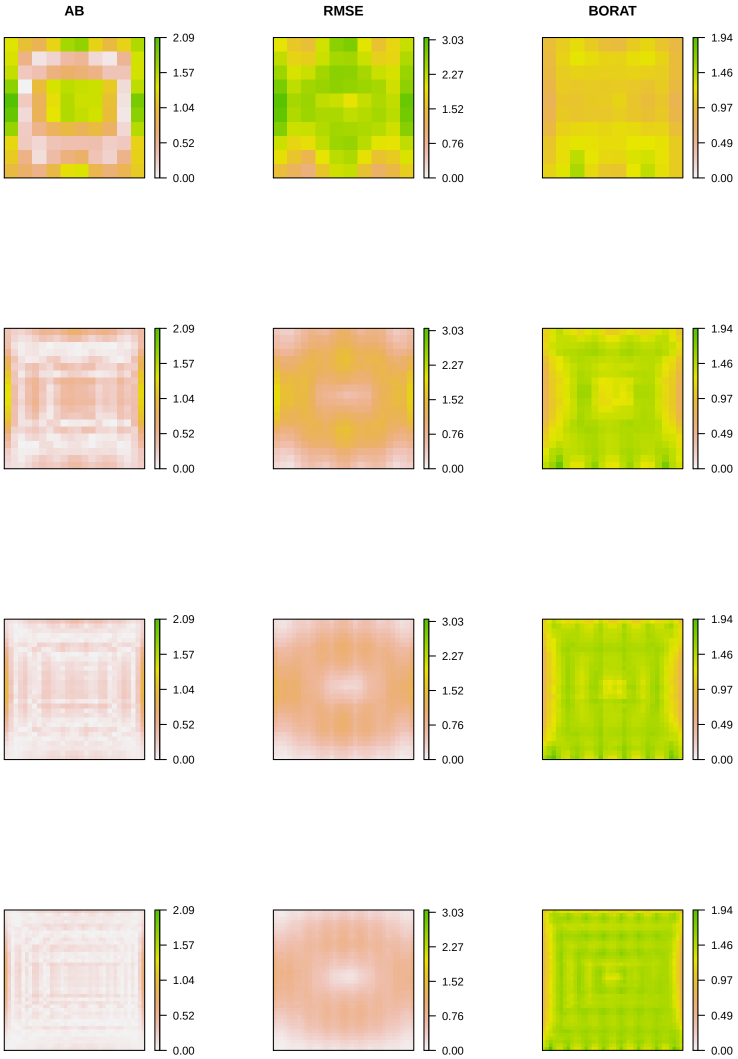

Web Figure 17: Maps of absolute bias (AB), root mean squared error (RMSE) and bootstrap ratio (BORAT) for populations of 100, 400, 900, 1600 quadrats from surface 2 under OPSS with 10% sampling fraction (rows 1-4 respectively).

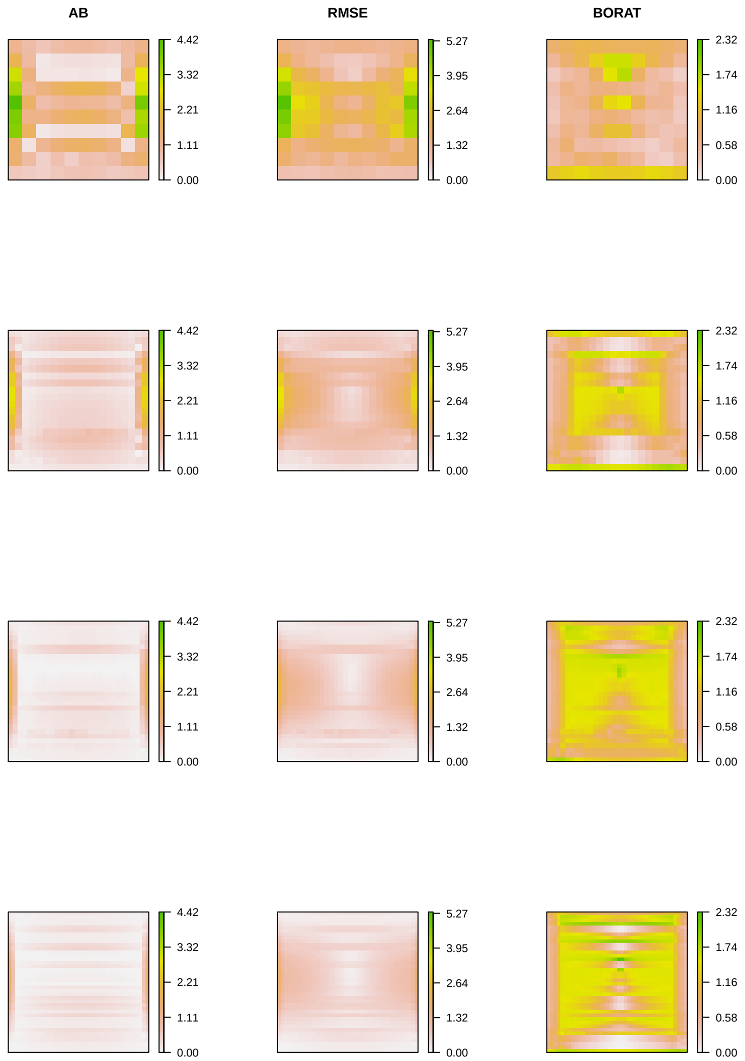

Web Figure 18: Maps of absolute bias (AB), root mean squared error (RMSE) and bootstrap ratio (BORAT) for populations of 100, 400, 900, 1600 quadrats from surface 2 under SYS with 10% sampling fraction (rows 1-4 respectively).

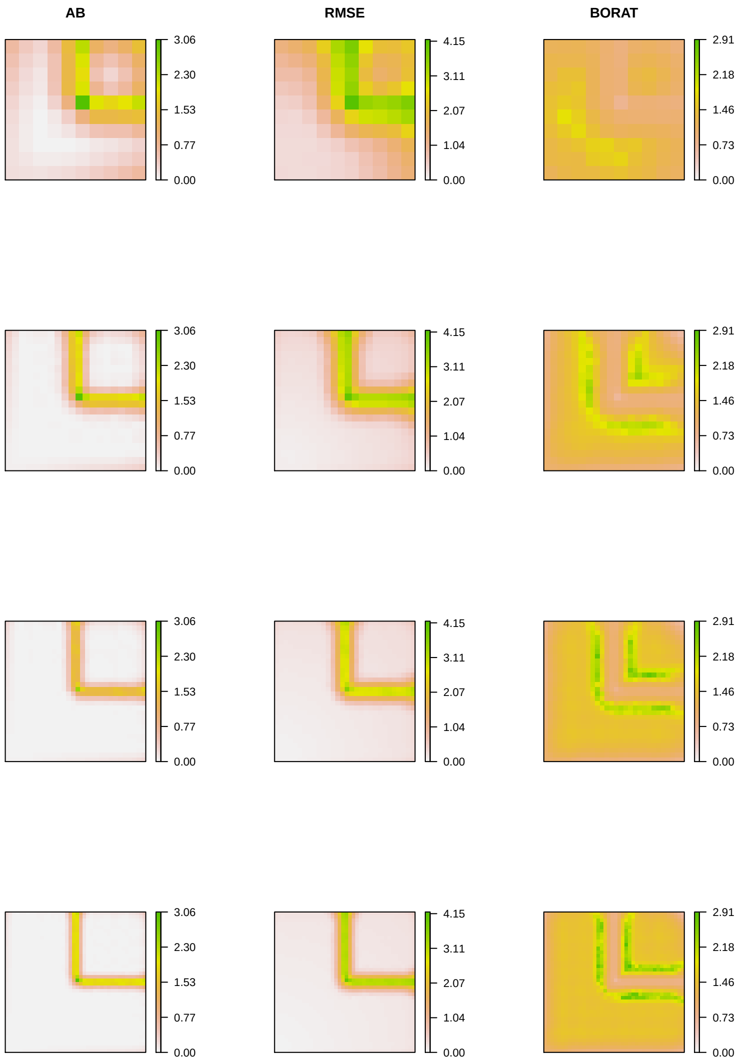

Web Figure 19: Maps of absolute bias (AB), root mean squared error (RMSE) and bootstrap ratio (BORAT) for populations of 100, 400, 900, 1600 quadrats from surface 3 under SRSWOR with 10% sampling fraction (rows 1-4 respectively).

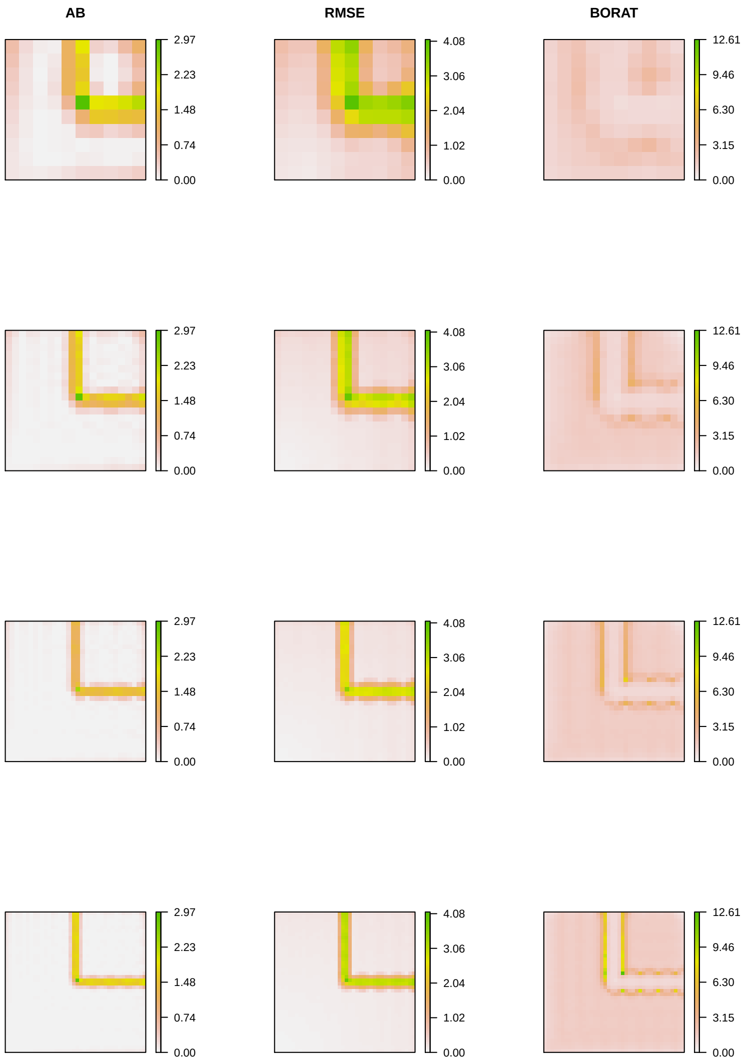

Web Figure 20: Maps of absolute bias (AB), root mean squared error (RMSE) and bootstrap ratio (BORAT) for populations of 100, 400, 900, 1600 quadrats from surface 3 under OPSS with 10% sampling fraction (rows 1-4 respectively).

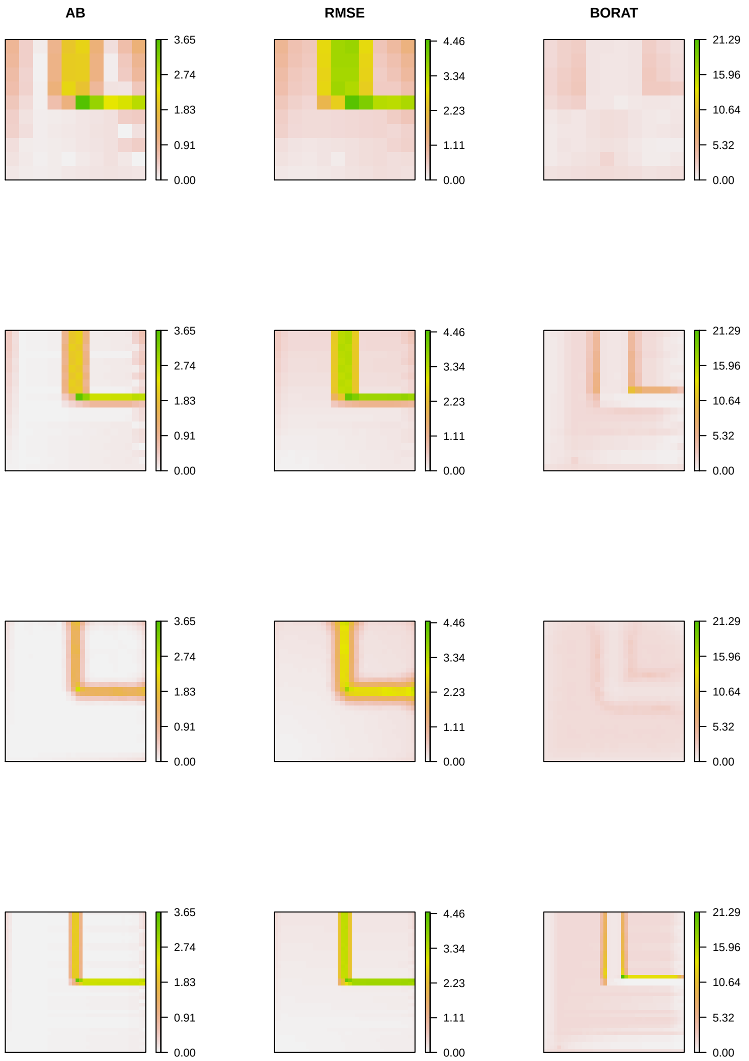

Web Figure 21: Maps of absolute bias (AB), root mean squared error (RMSE) and bootstrap ratio (BORAT) for populations of 100, 400, 900, 1600 quadrats from surface 3 under SYS with 10% sampling fraction (rows 1-4 respectively).

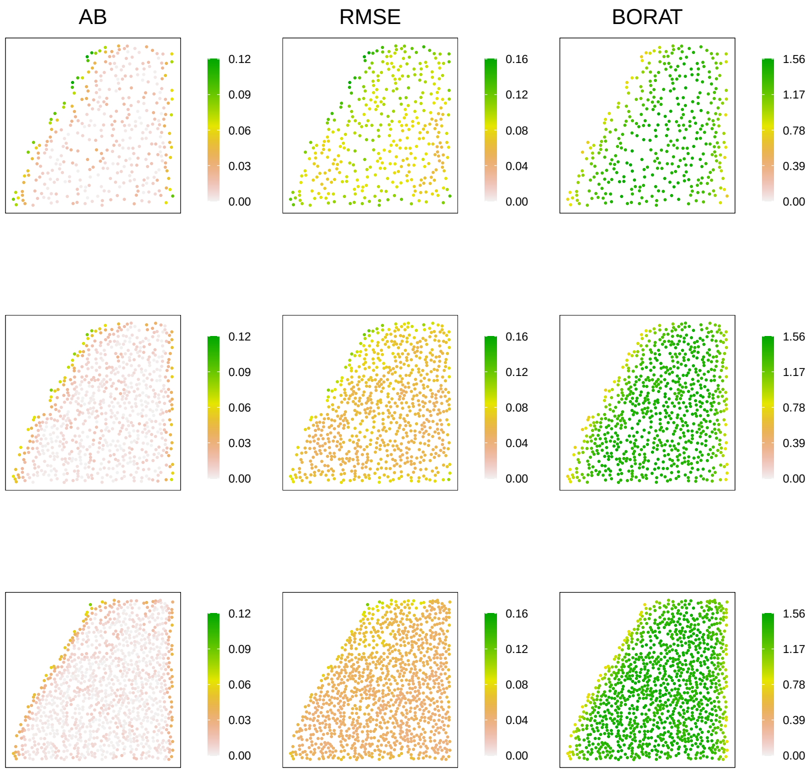

Web Figure 22: Maps of absolute bias (AB), root mean squared error (RMSE) and bootstrap ratio (BORAT) for nested populations of $358, 729, 1102$ units with regular pattern and having Y-values from 4 to 10 generated from surface 1 under 3P sampling with constant $L^* = 50$ (rows 1-3 respectively).

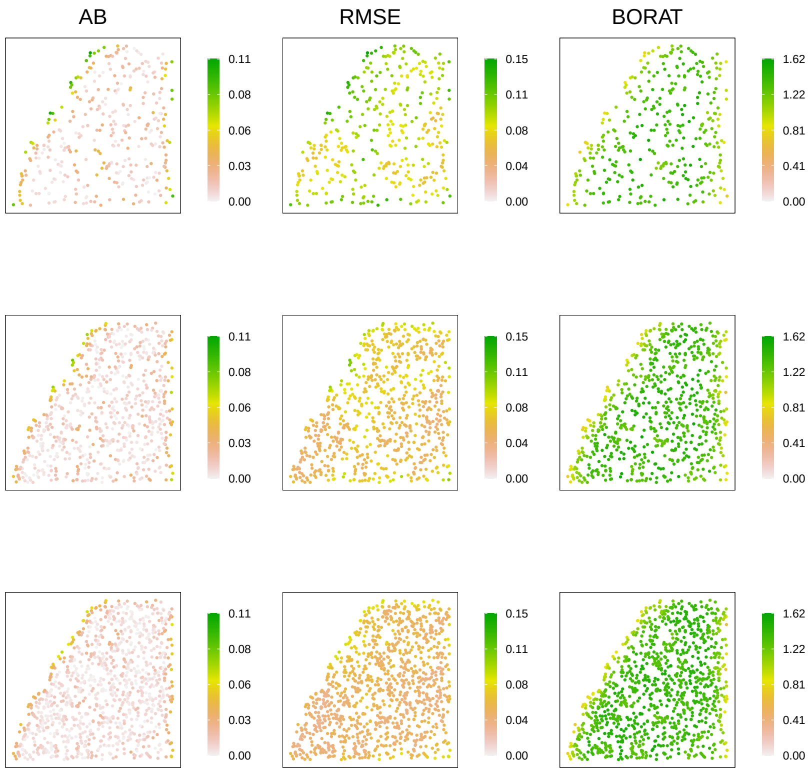

Web Figure 23: Maps of absolute bias (AB), root mean squared error (RMSE) and bootstrap ratio (BORAT) for nested populations of $349, 711, 1059$ units with random pattern and having Y-values from 4 to 10 generated from surface 1 under 3P sampling with constant $L^* = 50$ (rows 1-3 respectively).

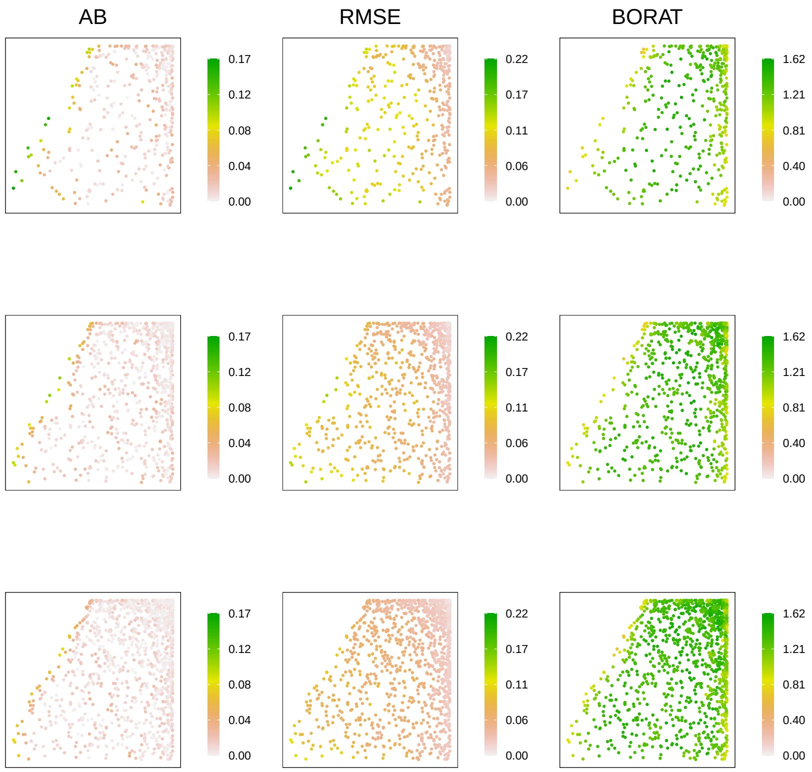

Web Figure 24: Maps of absolute bias (AB), root mean squared error (RMSE) and bootstrap ratio (BORAT) for nested populations of $404, 807, 1231$ units with trended pattern and having Y-values from 4 to 10 generated from surface 1 under 3P sampling with constant $L^* = 50$ (rows 1-3 respectively).

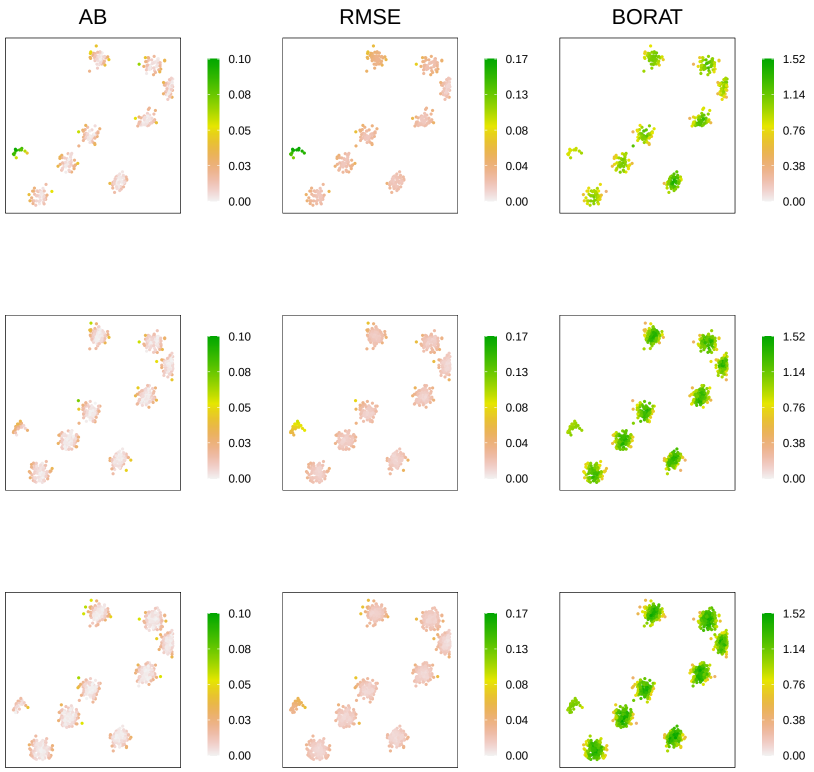

Web Figure 25: Maps of absolute bias (AB), root mean squared error (RMSE) and bootstrap ratio (BORAT) for nested populations of $413, 827, 1237$ units with clustered pattern and having Y-values from 4 to 10 generated from surface 1 under 3P sampling with constant $L^* = 50$ (rows 1-3 respectively).

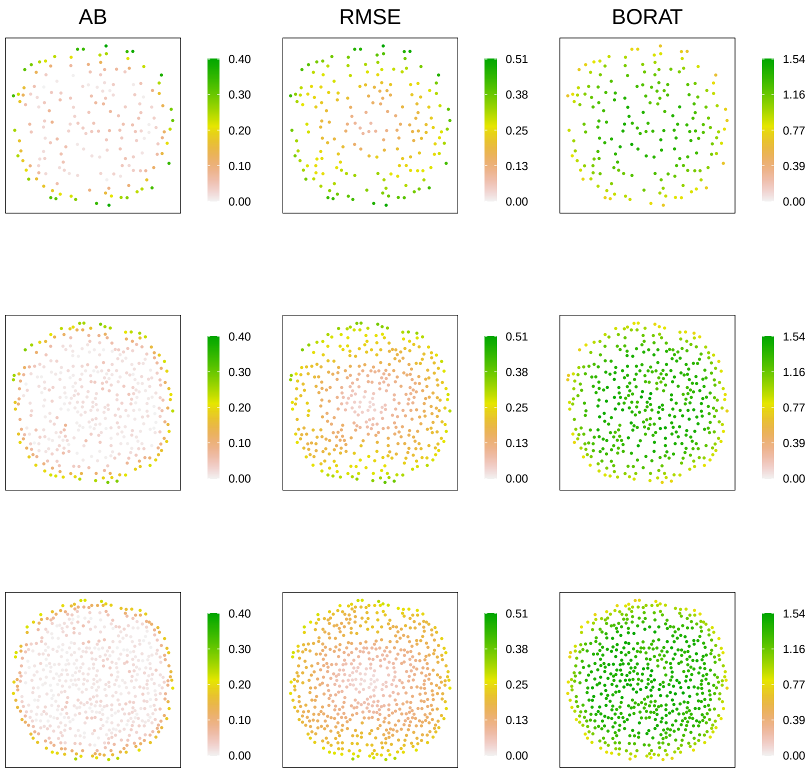

Web Figure 26: Maps of absolute bias (AB), root mean squared error (RMSE) and bootstrap ratio (BORAT) for nested populations of $179, 387, 579$ units with regular pattern and having Y-values from 4 to 10 generated from surface 2 under 3P sampling with constant $L^* = 50$ (rows 1-3 respectively).

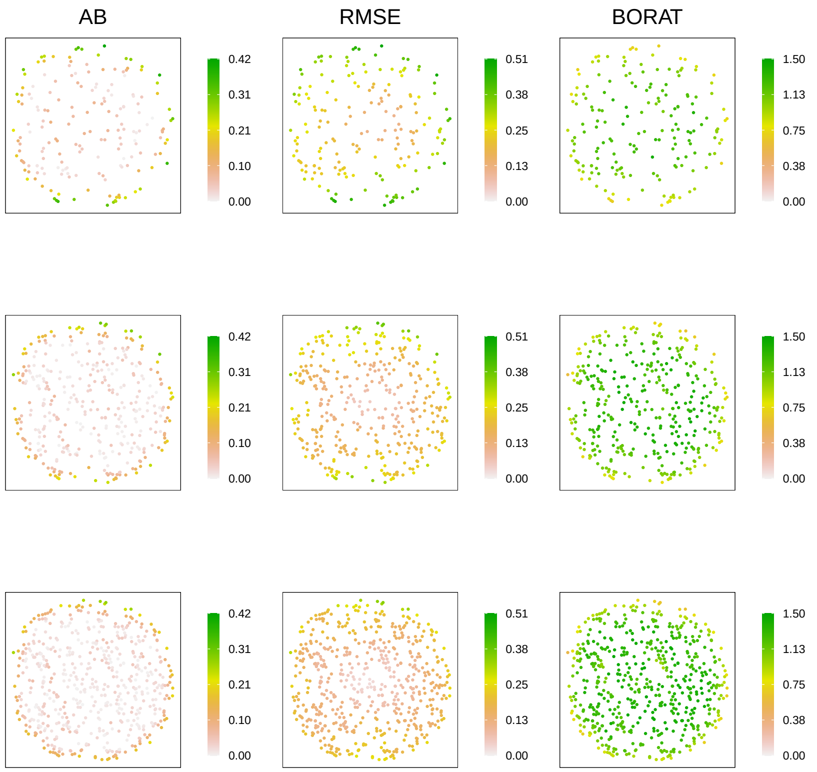

Web Figure 27: Maps of absolute bias (AB), root mean squared error (RMSE) and bootstrap ratio (BORAT) for nested populations of $179, 365, 571$ units with random pattern and having Y-values from 4 to 10 generated from surface 2 under 3P sampling with constant $L^* = 50$ (rows 1-3 respectively).

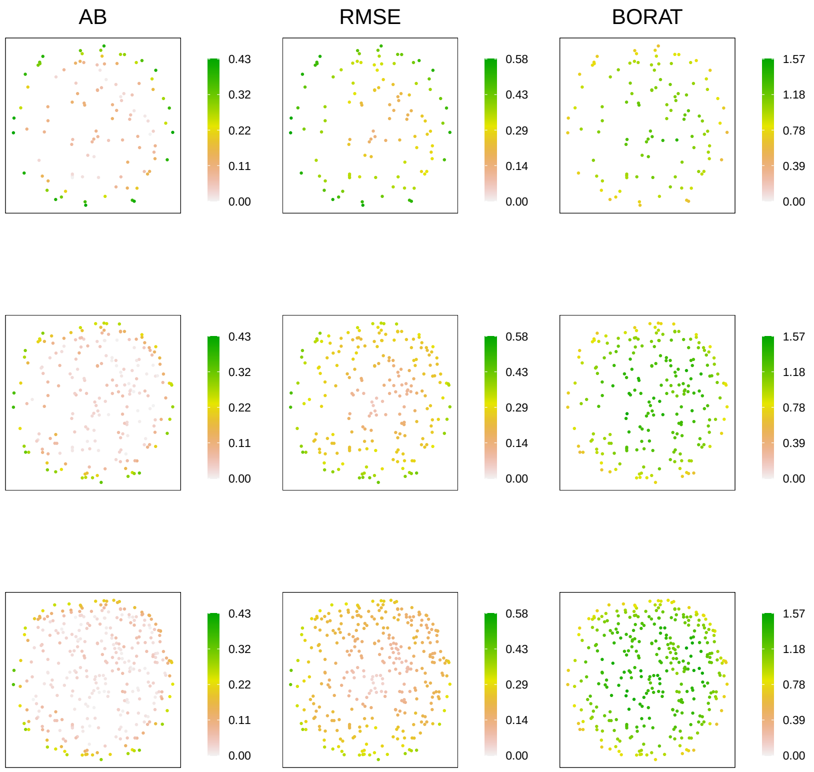

Web Figure 28: Maps of absolute bias (AB), root mean squared error (RMSE) and bootstrap ratio (BORAT) for nested populations of $105, 210, 327$ units with trended pattern and having Y-values from 4 to 10 generated from surface 2 under 3P sampling with constant $L^* = 50$ (rows 1-3 respectively).

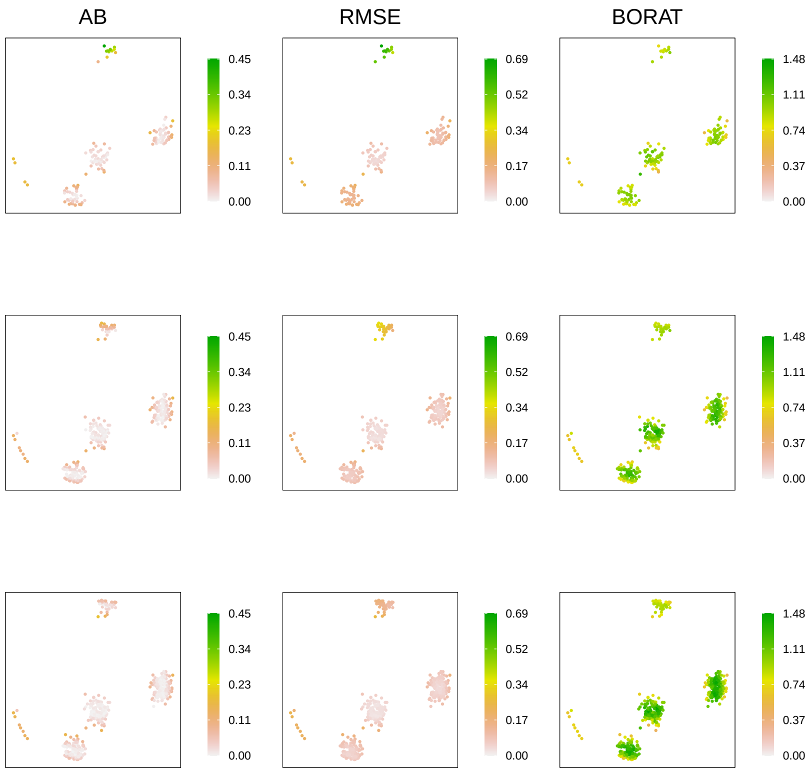

Web Figure 29: Maps of absolute bias (AB), root mean squared error (RMSE) and bootstrap ratio (BORAT) for nested populations of $154, 316, 473$ units with clustered pattern and having Y-values from 4 to 10 generated from surface 2 under 3P sampling with constant $L^* = 50$ (rows 1-3 respectively).

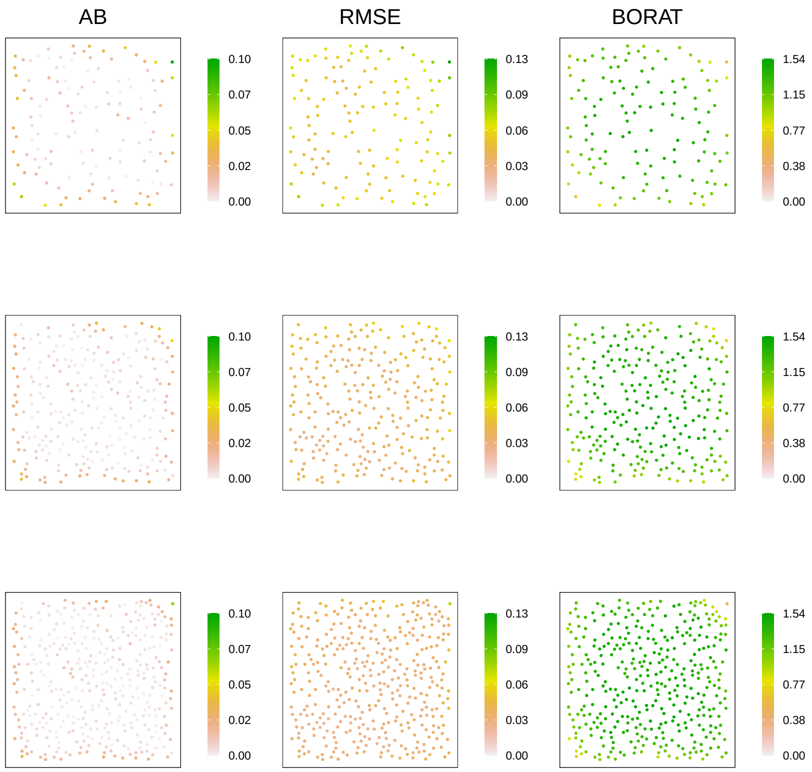

Web Figure 30: Maps of absolute bias (AB), root mean squared error (RMSE) and bootstrap ratio (BORAT) for nested populations of $131, 260, 373$ units with regular pattern and having Y-values from 4 to 10 generated from surface 3 under 3P sampling with constant $L^* = 50$ (rows 1-3 respectively).

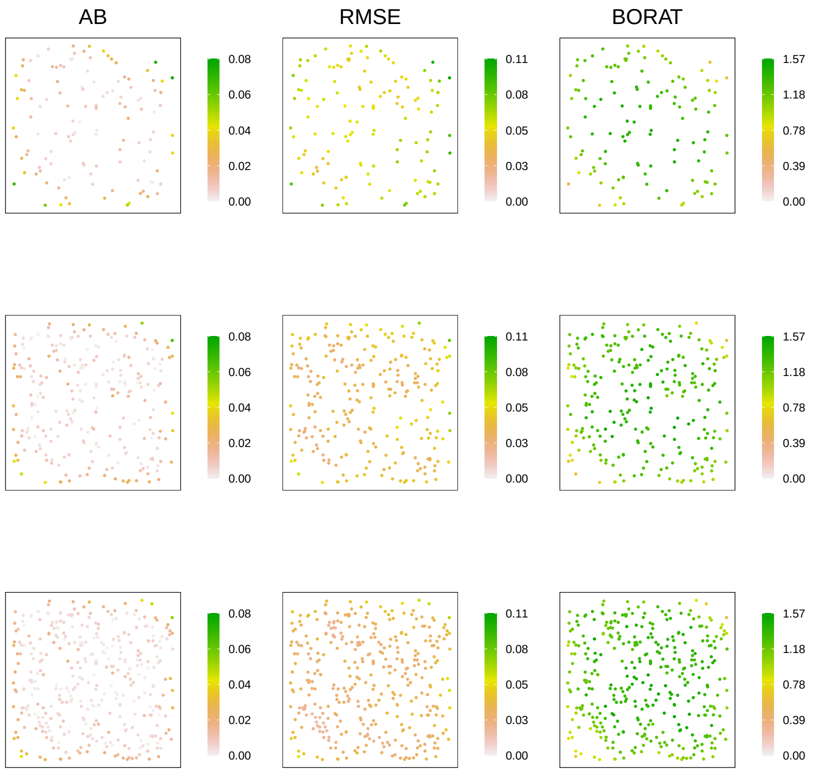

Web Figure 31: Maps of absolute bias (AB), root mean squared error (RMSE) and bootstrap ratio (BORAT) for nested populations of $124, 251, 366$ units with random pattern and having Y-values from 4 to 10 generated from surface 3 under 3P sampling with constant $L^* = 50$ (rows 1-3 respectively).

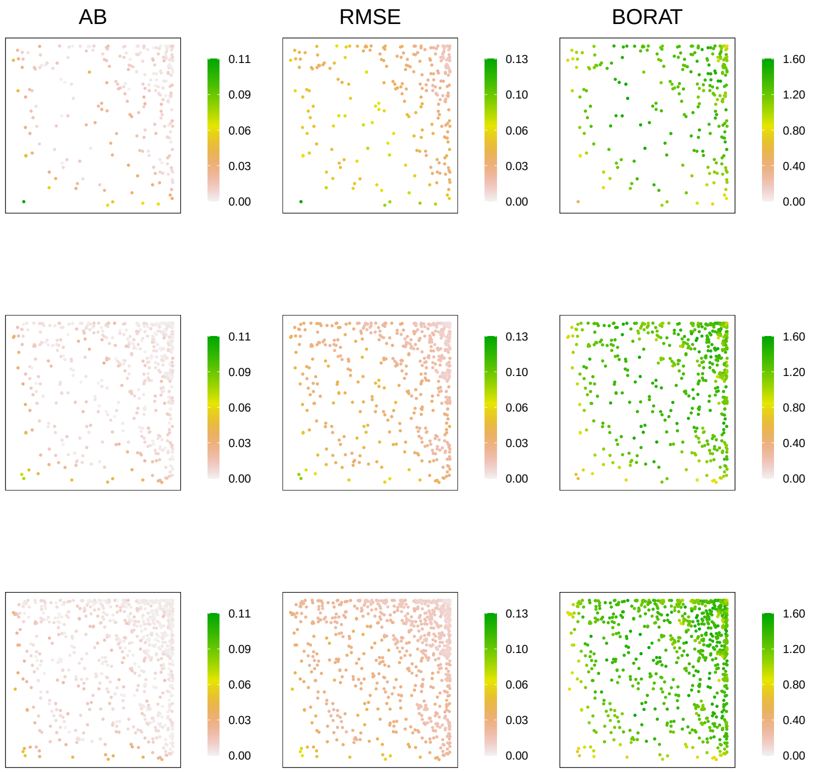

Web Figure 32: Maps of absolute bias (AB), root mean squared error (RMSE) and bootstrap ratio (BORAT) for nested populations of $251, 513, 783$ units with trended pattern and having Y-values from 4 to 10 generated from surface 3 under 3P sampling with constant $L^* = 50$ (rows 1-3 respectively).

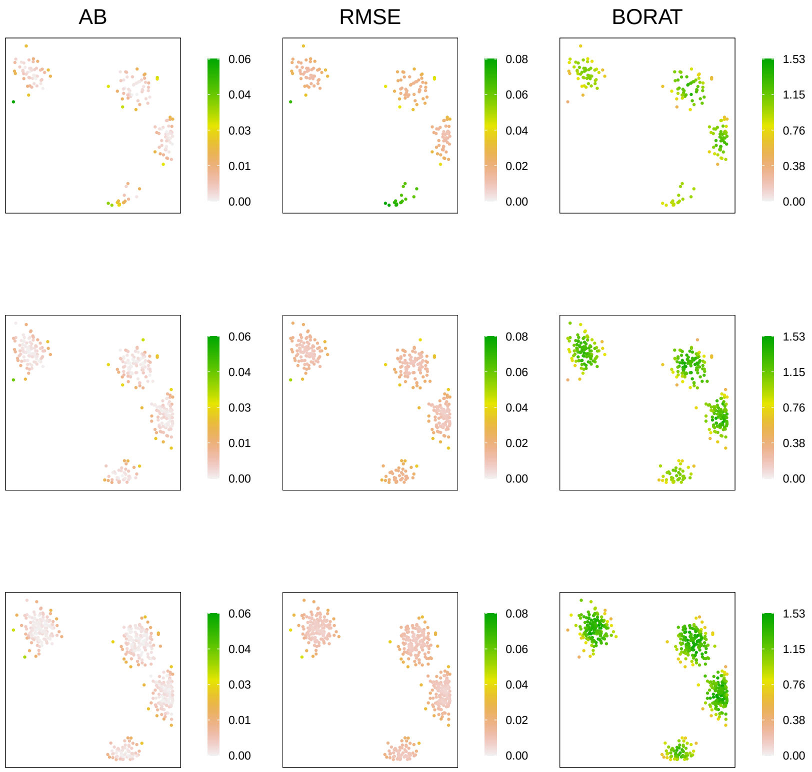

Web Figure 33: Maps of absolute bias (AB), root mean squared error (RMSE) and bootstrap ratio (BORAT) for nested populations of $166, 339, 511$ units with clustered pattern and having Y-values from 4 to 10 generated from surface 3 under 3P sampling with constant $L^* = 50$ (rows 1-3 respectively).
